# European Lampreys: New Insights on Postglacial Colonization, Gene Flow and Speciation

**DOI:** 10.1371/journal.pone.0148107

**Published:** 2016-02-12

**Authors:** Catarina Sofia Mateus, Pedro Raposo Almeida, Natacha Mesquita, Bernardo Ruivo Quintella, Maria Judite Alves

**Affiliations:** 1 MARE – Centro de Ciências do Mar e do Ambiente, Universidade de Évora, Évora, Portugal; 2 Museu Nacional de História Natural e da Ciência & Centro de Ecologia, Evolução e Alterações Ambientais, Universidade de Lisboa, Lisboa, Portugal; 3 Departamento de Biologia, Escola de Ciências e Tecnologia, Universidade Évora, Évora, Portugal; 4 Departamento de Biologia Animal, Faculdade de Ciências da Universidade de Lisboa, Lisboa, Portugal; Fordham University, UNITED STATES

## Abstract

Ice ages are known to be the most dominant palaeoclimatic feature occurring on Earth, producing severe climatic oscillations and consequently shaping the distribution and the population structure of several species. Lampreys constitute excellent models to study the colonization of freshwater systems, as they commonly appear in pairs of closely related species of anadromous *versus* freshwater resident adults, thus having the ability to colonize new habitats, through the anadromous species, and establish freshwater resident derivates. We used 10 microsatellite loci to investigate the spatial structure, patterns of gene flow and migration routes of *Lampetra* populations in Europe. We sampled 11 populations including the migratory *L*. *fluviatilis* and four resident species, *L*. *planeri*, *L*. *alavariensis*, *L*. *auremensis* and *L*. *lusitanica*, the last three endemic to the Iberian Peninsula. In this southern glacial refugium almost all sampled populations represent a distinct genetic cluster, showing high levels of allopatric differentiation, reflecting long periods of isolation. As result of their more recent common ancestor, populations from northern Europe are less divergent among them, they are represented by fewer genetic clusters, and there is evidence of strong recent gene flow among populations. These previously glaciated areas from northern Europe may have been colonized from lampreys expanding out of the Iberian refugia. The pair *L*. *fluviatilis/L*. *planeri* is apparently at different stages of speciation in different locations, showing evidences of high reproductive isolation in the southern refugium, and low differentiation in the north.

## Introduction

The Quaternary climatic oscillations and geographic restrictions imposed by the impassable glaciated areas are thought to have had major effects on the evolution and dispersal of various species (e.g. [1,2]). It is now clear that most fauna and flora presently distributed across Europe were isolated in southern refugia during the glacials, many in the Mediterranean peninsulas of Iberia, Italy and the Balkans [3]. After the glacials, and as the climate warmed rapidly, founder populations at the northern limits of the southern refugia expanded northwards, into the new available habitats, leading to a reduction from southern to northern Europe in the extent of the number of species, subspecific differentiation and allelic variation [3].

Recently deglaciated regions were relatively inaccessible to freshwater fishes, as they do not normally disperse among river basins; they were, however, easily reached by anadromous fishes (migratory species that reproduce in freshwater before migrating to the sea where they grow to the adult stage), which could access isolated basins *via* sea. These fish breed in fresh water, having ample opportunities to colonize these unexploited systems and establish freshwater isolates [4]. In some genera of lampreys, closely related species show divergent life histories: parasitic and anadromous vs. non-parasitic and freshwater resident; these species are called “paired species”, and the freshwater resident (brook) species have apparently evolved from a form similar to that of an extant anadromous one [5,6]. In some cases, more than one freshwater resident species has derived from a single anadromous species; these are called “satellite species” [7]. For this reason, lampreys constitute excellent systems to study the postglacial colonization processes and emergence of freshwater derivates from the founder anadromous forms.

The anadromous European river lamprey (*Lampetra fluviatilis*) and the resident European brook lamprey (*Lampetra planeri*) are considered paired species. They only occur in European watersheds, extending from southern Norway to the western Mediterranean and the Iberian Peninsula in the south. The three brook lampreys *Lampetra alavariensis*, *Lampetra auremensis* and *Lampetra lusitanica*, which are endemic to western Iberian Peninsula (Portugal) [8], are apparently derived from an extinct anadromous form, more ancestral than the solely extant anadromous form occurring in Iberia, *L*. *fluviatilis* [9]. In this region, while *L*. *planeri* is found in several river basins, *L*. *alavariensis*, *L*. *auremensis* and *L*. *lusitanica* are confined to one or two basins, and the anadromous *L*. *fluviatilis* currently occurs in the Tagus river basin only [8,10]. The current distribution of the extant Iberian lamprey lineages is largely allopatric and the genetic divergence between them is consistent with extended periods of isolation during survival in separate glacial refugia throughout the ice ages [9,11]. The three brook lampreys *L*. *alavariensis*, *L*. *auremensis* and *L*. *lusitanica* are well supported monophyletic groups, divergent from the present-day *L*. *fluviatilis*. However, *L*. *planeri* share haplotypes with the parasitic form, implying that their emergence was more recent [9,11]. The taxonomy of *L*. *fluviatilis* and *L*. *planeri* has thus been considered problematic, as studies using different markers have revealed lack of differentiation between the species (e.g. [11–13]), leaving open two possible scenarios: 1) a very recent divergence event or 2) a single species with phenotypic plasticity. The recent study of Mateus *et al*. [14], using genome-wide sequencing in sympatric populations of these species in the Iberian Peninsula, represented an important step forward in this long-standing question, as it successfully identified fixed allelic differences between the two forms in this region. These findings imply that these species are result of a recent divergence event (scenario 1), with populations in different phases of speciation across their ranges.

In this study, we investigate postglacial colonization of European fresh waters by analyzing patterns of genetic differentiation in a group with alternative life histories. We analyzed 10 polymorphic microsatellite loci, coupled with two mitochondrial genes, in the anadromous *L*. *fluviatilis* and in four resident species, *L*. *planeri*, *L*. *alavariensis*, *L*. *auremensis* and *L*. *lusitanica*. We tested the hypotheses that the Iberia is a source for the northward recolonization by the migratory species, the possible existence of a speciation gradient in Europe, and test for contemporary gene flow among and within species.

## Materials and Methods

### Ethics Statement

This study was carried out in strict accordance with the recommendations present in the Guide for the Care and Use of Laboratory Animals of the European Union—in Portugal represented by the Decree-Law n°129/92, Portaria n°1005/92. By the time the experimental work took place, the University’s ethics committee only dealt with research involving humans. Nevertheless, since two authors have an official license for animal experimentation (Category C from FELASA), issued by the Veterinary National Authority (DGV), Portuguese Ministry of Agriculture and Sea, the experiments involving living animals were performed in accordance to international rules regarding animal welfare. Collection of samples in Portugal was carried out without euthanizing the specimens, using the collecting permits provided by the Institute for Nature Conservation and Forestry, I.P. (ICNF, IP). In Belgium and Germany sampling authorizations are provided by Gouvernement Wallon—*Travaux publics*, *Agriculture*, *Ruralité*, *Nature*, *Forêt et Patrimoine*, and the German Federal Agency for Nature Conservation, respectively. In Finland, the samples have been collected from commercial lamprey catches, so those animals were caught for human consumption, and no authorization was needed.

### Sampling and DNA extraction

**Fig 1:**
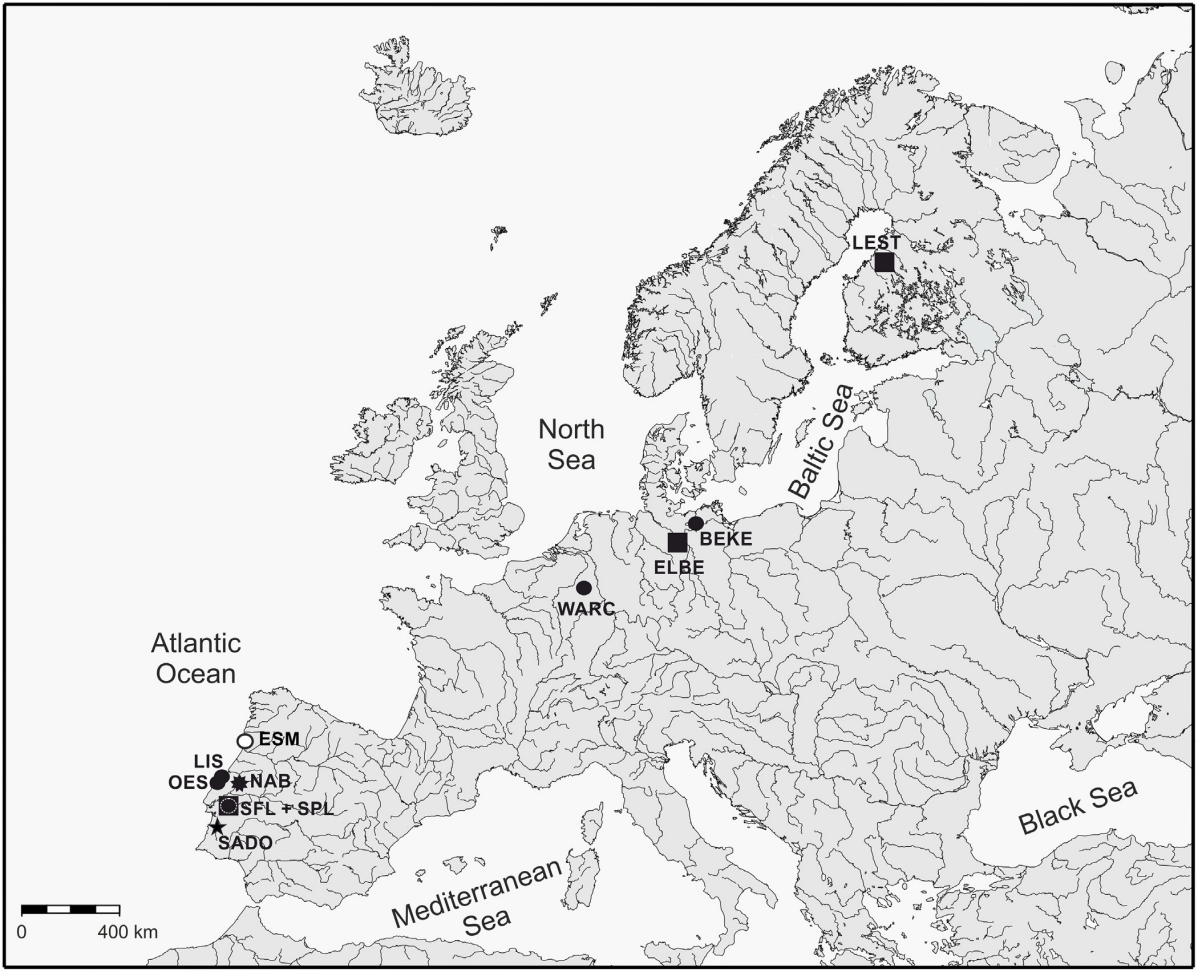
Sampling sites of *Lampetra* populations in Europe. Species: ■, *L*. *fluviatilis*; ●, *L*. *planeri*; ○, *L*. *alavariensis*; ★, *L*. *lusitanica*; 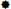, *L*. *auremensis*. See Table 1 for details about species and sampling sites.

Lampreys were collected by electro fishing, and after being anaesthetized by immersion in 2-phenoxyethanol (0.3 ml L^-1^), a piece of tissue was removed from the dorsal fin. After recovery individuals were released near the capturing sites. Sampled species were the European river lamprey *L*. *fluviatilis*, the European brook lamprey *L*. *planeri*, and the three recently described Iberian brook lampreys *L*. *alavariensis*, *L*. *auremensis* and *L*. *lusitanica* [8]. Ten sites were sampled, with only one species being present in each site, with the exception of the Sorraia River in the Tagus Basin (Iberian Peninsula), where *L*. *fluviatilis* and *L*. *planeri* are found in sympatry (Table 1). In the tables and across the manuscript, acronyms are labelled so that patterns are more readily understood: acronyms followed by _*m*_ refer to the migratory species (*L*. *fluviatilis*), and by _*r*_ to resident species (the brook lampreys *L*. *planeri*, *L*. *alavariensis*, *L*. *auremensis* and *L*. *lusitanica*). When treated together, populations from Belgium, Germany and Finland are hereinafter referred as “northern populations” and populations from the Iberian Peninsula as “southern populations”. All rivers sampled in the Iberian Peninsula drain to the Atlantic Ocean, rivers Warche (Meuse basin) and Schaale (Elbe basin) drain to the North Sea, whereas rivers Beke (Warnow basin) and Lestijoki drain to the Baltic Sea (Fig 1).

**Table 1:** Locations listed north to south, acronyms and sample sizes (*n*) of *Lampetra* samples included in the study.

Country	Basin	River	Acronym	*n*	Species
Finland	Lestijoki	Lestijoki	LEST_*m*_	29	*L*. *fluv*
Germany	Warnow	Beke	BEKE_*r*_	30	*L*. *plan*
Germany	Elbe	Schaale	ELBE_*m*_	40	*L*. *fluv*
Belgium	Meuse	Warche	WARC_*r*_	35	*L*. *plan*
Portugal	Esmoriz	Esmoriz	ESM_*r*_	33	*L*. *alavar*
Portugal	Lis	Lis	LIS_*r*_	33	*L*. *plan*
Portugal	Ribeiras do Oeste	Ribeira de S. Pedro	OES_*r*_	31	*L*. *plan*
Portugal	Tagus	Nabão	NAB_*r*_	35	*L*. *aurem*
Portugal	Tagus	Sorraia^1^	SPL_*r*_	52	*L*. *plan*
Portugal	Tagus	Sorraia^1^	SFL_*m*_	46	*L*. *fluv*
Portugal	Sado	Marateca	SADO_*r*_	51	*L*. *lusit*

Species: *L*. *plan*, *L*. *planeri*; *L*. *fluv*, *L*. *fluviatilis; L*. *alavar*, *L*. *alavariensis; L*. *aurem*, *L*. *auremensis; L*. *lusit*, *L*. *lusitanica*

Acronyms followed by _*m*_ refer to migratory species (*L*. *fluviatilis*), and by _*r*_ to resident species (*L*. *planeri*, *L*. *alavariensis*, *L*. *auremensis* and *L*. *lusitanica*).

^1^Location where the paired *L*. *fluviatilis* and *L*. *planeri* occur in sympatry.

Total genomic DNA was extracted following a standard phenol-chloroform protocol [15] and stored at -20°C. DNA concentration was measured using a Thermo Scientific NanoDrop^™^ 1000 Spectrophotometer and standardized to 50 ng μl^-1^ per sample.

### Microsatellite analysis

A total of 415 specimens from 10 sites were used in the analysis, with sample sizes ranging from 29 to 52 (Table 1). Initially, 49 microsatellite primer sets developed for other lamprey species (*Petromyzon marinus*: [16,17]; *Lethenteron* sp. N: [18]; *Ichthyomyzon unicuspis* and *Ichthyomyzon fossor*: [19]; *Lampetra richardsoni*: [20] were screened using the described protocols and were further optimized to the target species. Ten primer sets produced unambiguously determined bands and were polymorphic: Iun 2, Iun 5, Iun 7, Iun 10 and Iun 14 [19]; Lspn 010–2, Lspn 019c, Lspn 044 and Lspn 094 [18]; and Pma*μ* 5 [16]. These 10 loci were used to genotype 415 individuals; all others were rejected. The reverse primers were 5’-labelled with 6-FAM, NED, PET or VIC (Applied Biosystems^®^) fluorescent dyes. Primer sets were grouped into three multiplex reactions (S1 Table), and polymerase chain reactions (PCR) were set up in 12 μl volumes containing 2 μL of 50 ng μl^-1^ genomic DNA, 1.0 to 3.0 mM MgCl_2_, 0.2 mM dNTP mix, 0.5 μM for each primer, 1 unit of DreamTaq^™^ DNA Polymerase (Fermentas) and 1× DreamTaq^™^ Buffer. PCR conditions were as follows: initial denaturation at 94°C for 1 min, followed by 25 cycles of 30 sec at 94°C, annealing for 30 sec at temperatures ranging from 55 to 60°C and 30 sec at 72°C, and a final extension of 7 min at 72°C. A number of sets of difficult amplification were completed using a Multiplex PCR Kit (Qiagen^®^) with 5 μl Qiagen Multiplex PCR master Mix, 3 μl RNase-free water, 1 μl Primer Mix (2 μM each primer) and 1 μl of 50 ng μl^-1^ of genomic DNA, using the following protocol: initial activation step at 95°C for 15 min, followed by 30 cycles of denaturation at 94°C for 30 sec, annealing at 57°C for 90 sec and extension at 72°C for 60 sec, and a final extension of 30 min at 60°C. The PCR reactions were conducted on a Bio-Rad^®^ thermal cycler.

Samples were genotyped in an ABI PRISM^®^ 310 Genetic Analyzer and fragments were sized with GeneScan™-500 LIZ™ Size Standard. Allele sizes were determined using the software GeneMapper^®^ 3.7 (Applied Biosystems^®^).

Microsatellite loci were first tested for null alleles, stuttering and large allele dropout using the software MICRO-CHECKER 2.2.3 [21]. Each microsatellite locus was tested for Hardy–Weinberg equilibrium and departures from linkage equilibrium were assessed for all pairs of loci in each population with 10^4^ permutations, as implemented in ARLEQUIN 3.11 [22]. Genetic diversity was measured as the mean allelic richness (AR), observed heterozygosity (Ho), unbiased expected heterozygosity (He, *sensu* Nei 1978) and mean number of alleles across loci (MNA), inferred using GENETIX 4.05.2 [23], with the exception of allelic richness, which was calculated and corrected for sample dimension by rarefaction using HP-Rare [24].

The genetic differentiation among samples was accessed through pairwise *F*_ST_ using the Weir & Cockerham's estimator [25], and significance was assessed with 10^4^ permutations, as implemented in GENETIX. The distribution of genetic variation was accessed among and within the 11 samples, the sympatric *L*. *fluviatilis* and *L*. *planeri*, and the genetic clusters attained with population structure analysis, through analysis of molecular variance (AMOVA) [26]. These analyses were performed in ARLEQUIN, using the allelic frequencies as the genetic distance and 10^4^ permutations.

The Bayesian model-based clustering approach implemented in STRUCTURE 2.2 [27] was used to assemble individuals into groups (genetic clusters). Runs were performed under the admixture model, with correlated allelic frequencies, and with a number of groups (K) set between 1 and 12. For each K, 10 simulations were performed with a burn-in period of 10^5^, followed by 5 million Markov steps. Using the same parameters, two additional structure analyses were performed, one including solely the eight samples of *L*. *planeri* and *L*. *fluviatilis* (K between 1 and 9), and the other including the four samples from the North (K between 1 and 5). These allow detection of further structure in these populations, if present, that otherwise would be hidden due to the high differentiation among the five species, and between the northern and southern samples. The optimal K, and clustering achieved, was inferred using the protocol defined by Evanno *et al*. [28] as implemented in STRUCTURE HARVESTER 0.6.93 [29], and taking into account the biological meaning of the clusters. The software DISTRUCT 1.1 [30] was used for the graphical display of population clusters.

The software GeneClass2 2.0.h [31] was used to test the assignment of individuals to their sampling sites, including a likelihood-based method in which individuals are assigned to the locality in which the individual’s genotype is most likely to occur. The Bayesian statistical approach of Rannala & Mountain [32] was implemented. GeneClass2 2.0.h was also used to determine whether our samples might contain individuals that were first generation (F_0_) immigrants from unsampled populations (the so-called ‘ghost populations’, [33]). We used the Bayesian assignment procedure of Rannala & Mountain [32], and the Paetkau *et al*. method [34] to compute probabilities from 10,000 simulated genotypes.

Patterns of differentiation were visualized by principal coordinates analysis (PCoA), a multivariate technique that allows to find and plot the major patterns within a multivariate dataset, like multiple loci and multiple samples. This analysis was computed using GenAlEx 6.5 [35,36].

The software NewHybrids 1.1 [37] was used for the detection and classification of putative hybrids between sympatric populations of *L*. *fluviatilis* and *L*. *planeri* from Portugal. NewHybrids uses a Bayesian approach to identify different categories of hybrid individuals through the computation of the posterior probability that individuals fall into different hybrid (F_1_, F_2_ and backcrosses) or pure parental categories. It uses the allele frequencies of multilocus genotypes and a Markov Chain Monte Carlo procedure. Simulations were performed with a burn-in period of 10^5^, followed by a sampling period of 10^5^ Markov steps. A threshold of posterior probability > 50% was set up to classify an individual as belonging to a certain category.

Estimates of recent migration rates (*m*) between migratory populations were inferred using a Bayesian assignment test-based method in the program BAYESASS 3.0.1 [38]. We also performed migration rate analysis between all populations, whose results are presented in S2 Table. BAYESASS estimates migration rates over the last two generations using a Markov chain Monte Carlo procedure and does not assume that populations are in migration-drift or Hardy–Weinberg equilibrium. Because BAYESASS focuses on contemporary migration rates, estimates are unaffected by the colonization processes. A total of 10^7^ MCMC iterations (discarding the first 10^6^ iterations as burn-in) were performed, and samples were collected every 2000 iterations. The convergence and stability of the MCMC algorithm was checked by visual inspection of plotted posterior parameter estimates using the software Tracer 1.6 [39] (S1 Fig). Delta values for migration rate, allele frequencies, and inbreeding values coefficients were set at 0.20, 0.40 and 0.60, respectively.

Demographic signatures of recent bottlenecks were tested using the heterozygosity excess method implemented in BOTTLENECK 1.2.02 [40] under three different mutational models: infinite allele model (IAM), stepwise mutation model (SMM) and two-phase model (TPM). Significant deviations from mutational-drift equilibrium were tested using the Wilcoxon sign rank test with 10^5^ simulations, and the distribution of allele frequency classes was examined for a deviation from the normal L-shaped distribution [41]. Past reductions in population size were also evaluated using the *M* ratio (*M* = *k*/*r*) statistic test as implemented in M_P_VAL [42], where *k* is the number of alleles present at a given microsatellite locus and *r* is the overall range in allele size. In recently reduced populations *M* is expected to be smaller than in populations at equilibrium, since the loss in any allele will contribute to a reduction in *k*, whereas only a loss of the smallest or largest alleles will contribute to a reduction in *r*, and thus *k* is expected to decrease more quickly than *r*. Reductions in population size were considered significant if less than 5% of the replicates are below the observed *M* value. Following Garza & Williamson (2001) [42], we used the default settings for the two-phase mutation model (TPM) *p*_s_ = 0.9, Δ_g_ = 3.5 and three values of *θ* (*θ* = 4, *θ* = 10 and *θ* = 20), where *θ* = 4Neμ (Ne = effective population size; μ = mutation rate), *p*_s_ is the proportion of one-step mutations, and Δ_g_ is the average size of non one-step mutations.

Populations LIS and OES were not included in the demographic analysis because they present only one polymorphic locus.

### MtDNA sequence analysis

For a better understanding of the historical processes that may underlie present population structure, we performed a phylogenetic analysis of mtDNA sequences of the populations included in this study, and additional populations from across the species distributional range.

A total of 44 samples from northern Europe (LEST_*m*_, BEKE_*r*_, ELBE_*m*_, WARC_*r*_) were amplified and sequenced for both cytochrome *b* and ATPase 6/8 mitochondrial genes, following the protocol in Mateus *et al*. [9]. For the remaining populations included in the microsatellite analysis, the corresponding haplotypes were obtained from the GenBank nucleotide data base [9,11], as well as additional haplotypes which represent other populations from across the distributional range of *L*. *planeri* and *L*. *fluviatilis*. MtDNA sequencing of 44 individuals from LEST_*m*_, BEKE_*r*_, ELBE_*m*_ and WARC_*r*_ revealed a total of 14 new haplotypes (H83 to H96), which combined with the 52 haplotypes from GenBank [9,11] resulted in a dataset of 66 haplotypes for phylogenetic analyses. New nucleotide sequences are available at the GenBank database under the accession numbers KT275288- KT275301.

DNA sequences were manually aligned and edited using Sequencher V4.8 (Gene Codes Corp., Ann Arbor, MI, USA). One sequence each of the species *L*. *alavariensis*, *L*. *auremensis* and *L*. *lusitanica* was included and used as outgroup (haplotypes H26, H48 and H53, respectively; [9,11]).

The phylogenetic relationships among mtDNA haplotypes were reconstructed using PAUP* [43] by the neighbour-joining algorithm [44], according to the selected model and using the parameter settings as estimated with MODELTEST software [45]. Bootstrap support values in neighbour-joining analysis were computed with fast stepwise addition using 1000 pseudo-replicates. For maximum parsimony, bootstrap values were computed with full heuristic search using 500 replicates, each consisting of 100 TBR searches of random addition sequence.

## Results

### Genetic diversity and differentiation

For microsatellites, signs of null alleles were detected with MICRO-CHECKER for a few situations across loci and populations: Pma*μ* 5 in SPL_*r*_ and NAB_*r*_; Iun 10 in SPL_*r*_ and BEKE_*r*_; Iun 5 in ELBE_*m*_ and BEKE_*r*_; and Iun 14 in SPL_*r*_ and BEKE_*r*_. Significant null alleles’ signature is related with heterozygote deficit and therefore with deviations from Hardy–Weinberg equilibrium, as seen in S2 Table.

The summary statistics of the genetic diversity indices for each locus and sample are provided in S2 Table. The total number of alleles per locus across populations varied from two, at the loci Iun7 and Lspn010-2, to 13 at the locus Iun14. Twelve private alleles were found: NAB_*r*_, SPL_*r*_ and SADO_*r*_ showed three unique alleles each, BEKE two, and SFL one (S2 Table). The mean number of alleles (MNA) across loci ranged from 1.1 (LIS_*r*_ and OES_*r*_) to 3.8 (ELBE_*m*_), mean allelic richness (AR) from 1.08 (LIS_*r*_) to 2.62 (ELBE_*m*_), and expected heterozygosity (He) from 0.0239 (LIS_*r*_) to 0.4417 (NAB_*r*_) (S2 Table).

A considerable level of genetic differentiation among samples was observed (average *F*_ST_ = 0.498, *P*<0.001) with pairwise *F*_ST_ values ranging from 0.0114 (ELBE_*m*_-LEST_*m*_) to 0.8915 (OES_*r*_-ESM_*r*_), all being significant (*P*<0.001 for all pairs, with the exception of ELBE_*m*_-LEST_*m*_ where *P*<0.05) (Table 2).

**Table 2:** Pairwise estimates of genetic differentiation (*F*_ST_) among sites (above diagonal) and corresponding *P* values (below diagonal). For populations’ acronyms, please check Table 1.

	LEST_*m*_	BEKE_*r*_	ELBE_*m*_	WARC_*r*_	ESM_*r*_	LIS_*r*_	OES_*r*_	NAB_*r*_	SPL_*r*_	SFL_*m*_	SADO_*r*_
**LEST**_***m***_	-	0.0519	0.0114	0.2849	0.5035	0.6642	0.6736	0.3820	0.2602	0.1463	0.4557
**BEKE**_***r***_	***	-	0.0530	0.3963	0.5301	0.6959	0.7104	0.3989	0.2919	0.2378	0.5243
**ELBE**_***m***_	0.04	***	-	0.2486	0.4644	0.6269	0.6402	0.3712	0.2330	0.1100	0.4127
**WARC**_***r***_	***	***	***	-	0.5692	0.7047	0.6974	0.4464	0.3408	0.3704	0.6056
**ESM**_***r***_	***	***	***	***	-	0.8877	0.8915	0.5472	0.3697	0.5131	0.7529
**LIS**_***r***_	***	***	***	***	***	-	0.6166	0.5820	0.5656	0.6897	0.8396
**OES**_***r***_	***	***	***	***	***	***	-	0.5839	0.5887	0.6996	0.8291
**NAB**_***r***_	***	***	***	***	***	***	***	-	0.3909	0.4423	0.6273
**SPL**_***r***_	***	***	***	***	***	***	***	***	-	0.3167	0.4931
**SFL**_***m***_	***	***	***	***	***	***	***	***	***	-	0.3989
**SADO**_***r***_	***	***	***	***	***	***	***	***	***	***	-

*** *P*<0.001.

**Fig 2:**
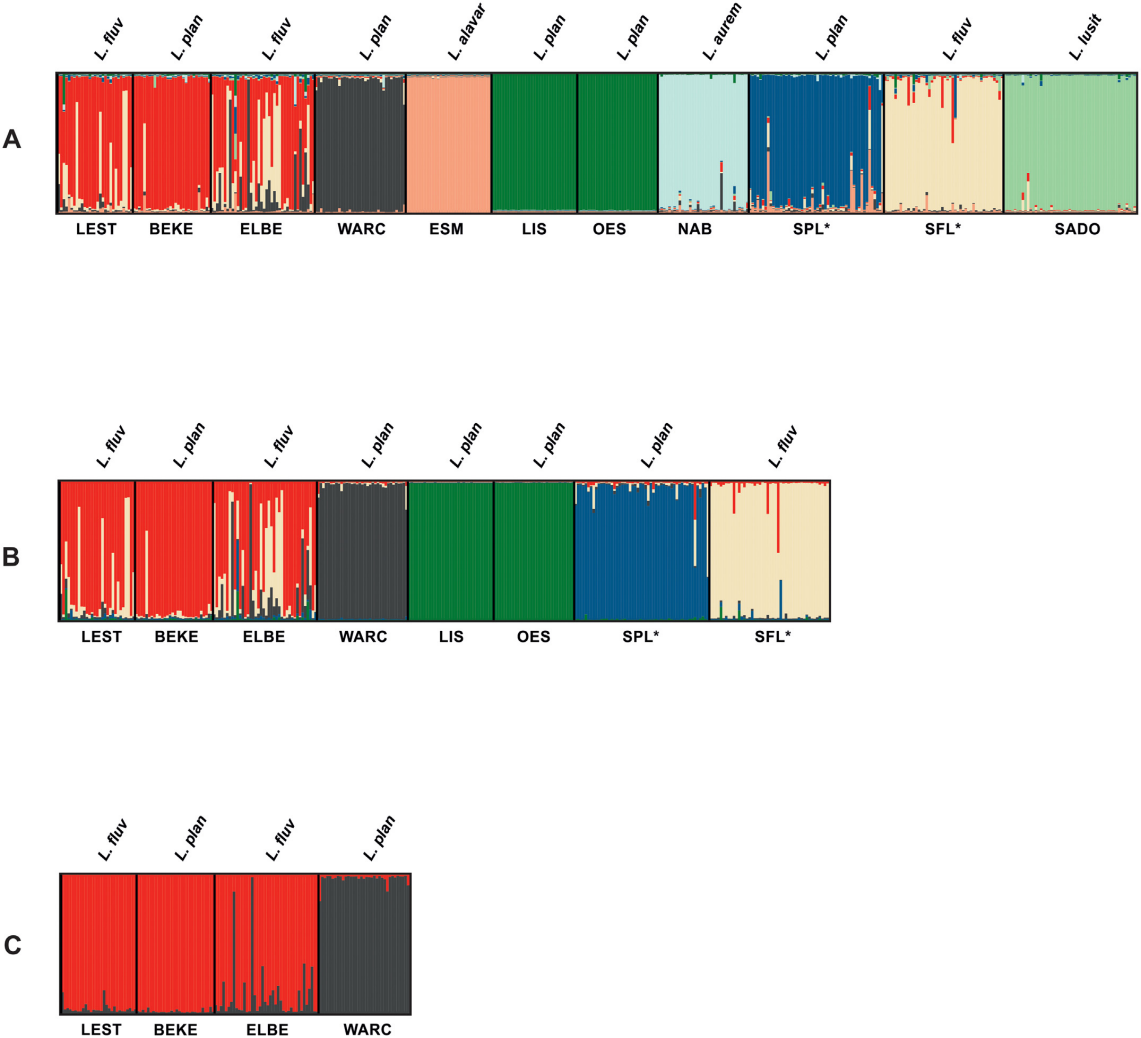
Most likely population structure, computed under the admixture model with correlated allelic frequencies in STRUCTURE, considering: A) all the 11 samples, K = 8; B) the populations of *L*. *planeri* and *L*. *fluviatilis*, K = 5; and C) the four northern populations, K = 2. Each individual is represented by a vertical bar. Sampled locations below plot and corresponding *Lampetra* species above are in accordance with Fig 1 and Table 1.

AMOVA analysis among the 11 samples indicated that 48.34% of genetic variance occurred among samples (*P*<0.001); AMOVA between the sympatric paired *L*. *fluviatilis*/*L*. *planeri* from the Tagus basin revealed that 31.64% of the variance was significantly (*P*<0.001) explained among species; and AMOVA among the eight genetic groups attained with STRUCTURE (see below and Fig 2A) indicated that the majority of variance occurs among groups (46.79%) and within samples (48.89%), whereas variance among samples within groups is low (4.32%) (*P*<0.001 for all the three levels).

### Population structure and admixture

The STRUCTURE analysis using the microsatellite data revealed that the 11 samples are grouped in eight distinct genetic clusters: 1) LEST_*m*_+BEKE_*r*_+ELBE_*m*_, 2) WARC_*r*_, 3) ESM_*r*_, 4) LIS_*r*_+OES_*r*_, 5) NAB_*r*_, 6) SPL_*r*_, 7) SFL_*m*_ and 8) SADO_*r*_ (Fig 2A and Table 3). The first group exhibits strong evidence of admixture with the resident *L*. *planeri* from Meuse (WARC_*r*_, group 2) and *L*. *fluviatilis* from Tagus (SFL_*m*_, group 7), with a greater number of admixed individuals in the anadromous populations (LEST_*m*_ and ELBE_*m*_) (Fig 2). Most individuals of group 2 (WARC_*r*_) are distinct and constitute a distinct genetic cluster with high proportion of membership (0.949; Table 3). This is also the case in other groups comprised of resident species, namely, ESM_*r*_, LIS_*r*_+OES_*r*_, NAB_*r*_ and SADO_*r*_ (groups 3, 4, 5, and 8, respectively; Fig 2A and Table 3). The sympatric paired *L*. *planeri* and *L*. *fluviatilis* from Tagus (SPL_*r*_ and SFL_*m*_) constitute two distinct genetic clusters (6 and 7, respectively) that present a few admixed individuals between them. SPL_*r*_ also shows some evidence of admixture with ESM_*r*_ (Fig 2A and Table 3). When STRUCTURE was run only for the eight samples of *L*. *planeri* and *L*. *fluviatilis* (Fig 2B, K = 5), and for the four northern samples (Fig 2C, K = 2), no additional genetic clusters were achieved, indicating that there is no hidden structure caused by the high differentiation of the Iberian endemics, and that the genetic cluster that groups the northern populations (LEST_*m*_+BEKE_*r*_+ELBE_*m*_) is well supported.

**Table 3:** STRUCTURE analysis for the 11 samples. Proportion of membership of each pre-defined population in each of the eight genetic clusters. For populations’ acronyms, please check Table 1.

Population	Inferred clusters							
	1	2	3	4	5	6	7	8
LEST_*m*_	**0.734**	0.028	0.015	0.013	0.007	0.010	0.189	0.005
BEKE_*r*_	**0.917**	0.008	0.017	0.006	0.008	0.006	0.032	0.007
ELBE_*m*_	**0.552**	0.135	0.016	0.012	0.014	0.017	0.242	0.011
WARC_*r*_	0.009	**0.949**	0.009	0.003	0.006	0.005	0.014	0.004
ESM_*r*_	0.003	0.003	**0.977**	0.003	0.003	0.004	0.005	0.003
LIS_*r*_	0.003	0.002	0.003	**0.981**	0.003	0.003	0.003	0.002
OES_*r*_	0.002	0.002	0.002	**0.983**	0.003	0.002	0.002	0.003
NAB_*r*_	0.008	0.018	0.009	0.007	**0.943**	0.006	0.005	0.003
SPL_*r*_	0.016	0.012	0.052	0.006	0.009	**0.860**	0.038	0.006
SFL_*m*_	0.035	0.009	0.016	0.007	0.004	0.016	**0.902**	0.012
SADO_*r*_	0.007	0.004	0.005	0.006	0.004	0.005	0.013	**0.957**

Clusters: 1, LEST_*m*_+BEKE_*r*_+ELBE_*m*_; 2, WARC_*r*_; 3, ESM_*r*_; 4, LIS_*r*_+OES_*r*_; 5, NAB_*r*_; 6, SPL_*r*_; 7, SFL_*m*_; 8, SADO_*r*_

Values in bold represent the proportion of membership in the dominant genetic cluster.

**Fig 3:**
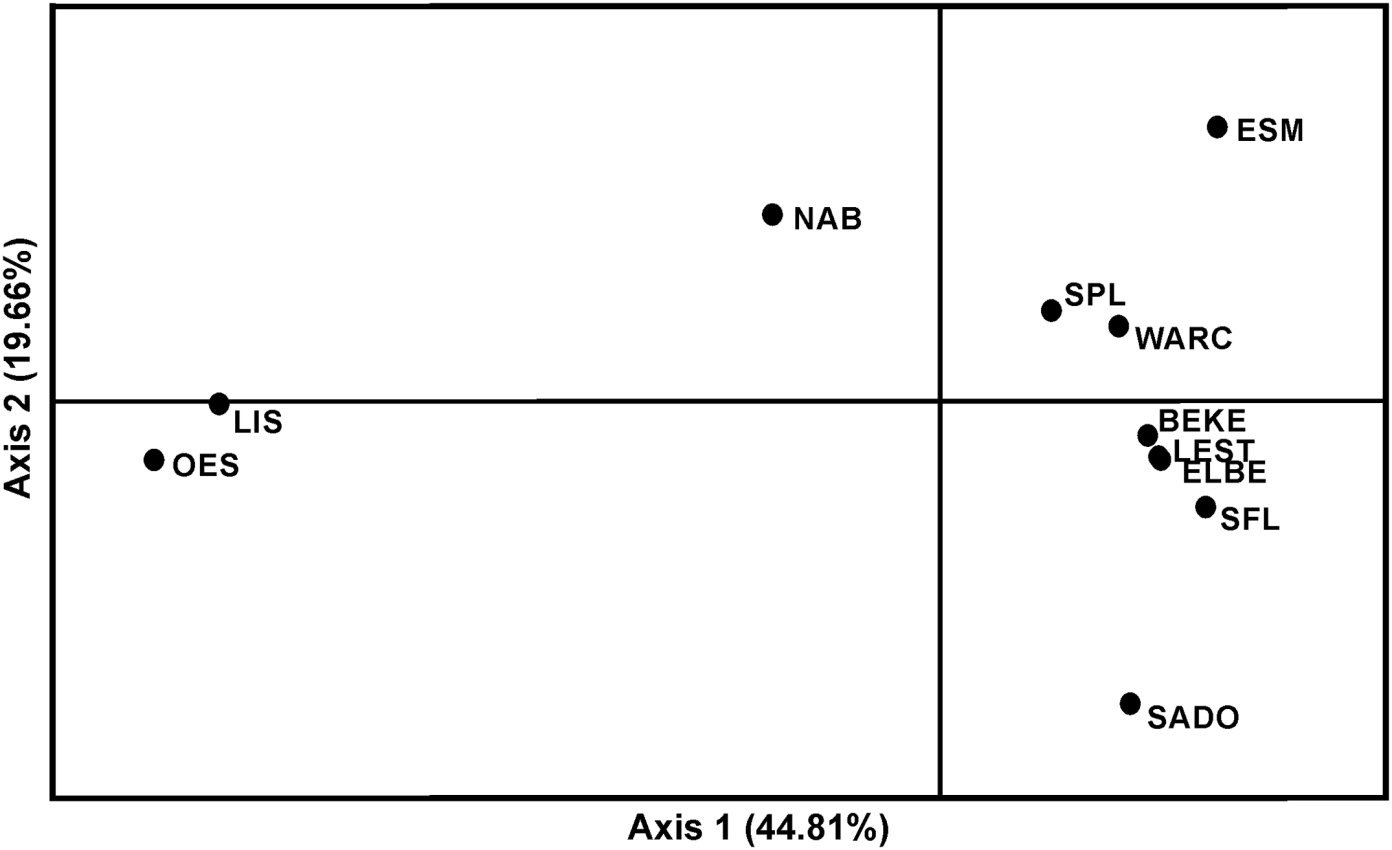
Principal coordinates analysis plot (PCoA) computed by GenAlEx. The percentage of variation explained by each axis is shown. Samples’ acronyms as in Fig 1 and Table 1.

Individual assignment tests were applied to further investigate the genetic distinctiveness of the populations. In four populations of resident species (WARC_*r*_, ESM_*r*_, NAB_*r*_ and SADO_*r*_) 100% of the individuals were assigned to their correct source population (Table 4), which is in agreement with the STRUCTURE analysis. Samples from northern Europe were the ones with more individuals assigned to other populations; *L*. *fluviatilis* from Lestijoki and Elbe (LEST_*m*_ and ELBE_*m*_, respectively) and the resident *L*. *planeri* from Warnow (BEKE_*r*_) had individuals assigned among the three populations, in agreement with STRUCTURE, in which the three populations form a distinct genetic cluster (see above). The sympatric *L*. *fluviatilis* and *L*. *planeri* (SFL_*m*_ and SPL_*r*_) had almost all individuals assigned correctly (96% and 98%), and small percentages (4% and 2%) assigned between them. LIS_*r*_ and OES_*r*_ showed 73% and 97%, respectively, of correctly assigned individuals, and the remaining were assigned also among each other (Table 4). This last result is consistent with the STRUCTURE and PCoA analyses, which revealed a close genetic relation between these two populations (Figs 2 and 3). Using a critical *P* of 0.01 for first-generation (F_0_) immigrant analysis, a total of seven individuals had a probability below the threshold value, but all were assigned to a sampled location: one individual of LEST_*m*_ was assigned to BEKE_*r*_; one of BEKE_*r*_ was assigned to ELBE_*m*_; one individual of ELBE_*m*_ was assigned to BEKE_*r*_ and another to WARC_*r*_; one individual of WARC_*r*_ was assigned to ELBE_*m*_; one individual of SPL_*r*_ was assigned to SFL_*m*_; and one individual of SFL_*m*_ was assigned to LEST_*m*_.

**Table 4:** Assignment tests performed with GeneClass2. Values represent the percentage of individuals from each studied sample assigned to each of the sampled populations based on the Bayesian method. For populations’ acronyms, please check Table 1.

	Assigned population										
	LEST_*m*_	BEKE_*r*_	ELBE_*m*_	WARC_*r*_	ESM_*r*_	LIS_*r*_	OES_*r*_	NAB_*r*_	SPL_*r*_	SFL_*m*_	SADO_*r*_
**LEST**_***m***_	**80**	3	17	0	0	0	0	0	0	0	0
**BEKE**_***r***_	3	**94**	3	0	0	0	0	0	0	0	0
**ELBE**_***m***_	10	15	**68**	5	0	0	0	0	0	2	0
**WARC**_***r***_	0	0	0	**100**	0	0	0	0	0	0	0
**ESM**_***r***_	0	0	0	0	**100**	0	0	0	0	0	0
**LIS**_***r***_	0	0	0	0	0	**73**	27	0	0	0	0
**OES**_***r***_	0	0	0	0	0	3	**97**	0	0	0	0
**NAB**_***r***_	0	0	0	0	0	0	0	**100**	0	0	0
**SPL**_***r***_	0	0	0	0	0	0	0	0	**98**	2	0
**SFL**_***m***_	0	0	0	0	0	0	0	0	4	**96**	0
**SADO**_***r***_	0	0	0	0	0	0	0	0	0	0	**100**

Each row contains the samples from one sampled locality and the columns indicate the localities to which the samples were assigned (*i*.*e*., in which their genotypes had the highest likelihood of occurring).

Values along the diagonal (in bold) represent the proportion of individuals assigned to the population in which they were sampled.

The principal coordinates analysis (PCoA), revealed the existence of mainly six distinct clusters, NAB_*r*_, SADO_*r*_, ESM_*r*_, LIS_*r*_+OES_*r*_, SPL_*r*_+WARC_*r*_, BEKE_*r*_+LEST_*m*_+ELBE_*m*_+SFL_*m*_ (Fig 3). These results are congruent with STRUCTURE, with the exception that it groups SPL_*r*_ with WARC_*r*_, and SFL_*m*_ with the northern cluster, while in STRUCTURE SPL_*r*_, WARC_*r*_, SFL_*m*_ and the northern populations BEKE_*r*_, LEST_*m*_ and ELBE_*m*_ constitute four distinct groups.

**Fig 4:**
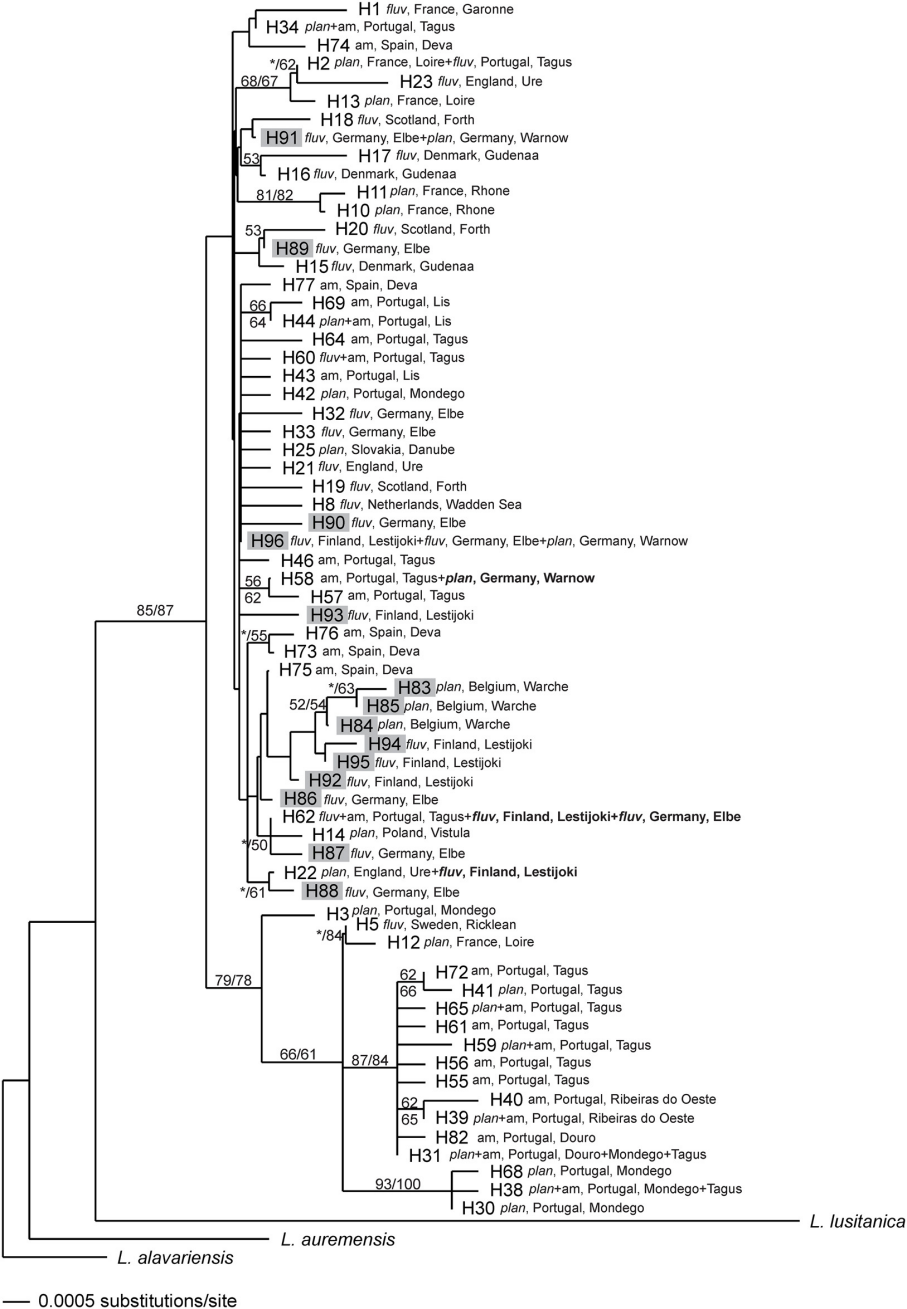
Neighbour-joining phylogenetic tree of 66 mitochondrial haplotypes of *Lampetra* (14 from this study (H83 to H96; grey shading) and the remaining 52 from GenBank database). For each haplotype, the species, country and river basin are indicated. Numbers are the bootstrap support values equal to or higher than 50% obtained from maximum parsimony (left or above) and neighbour-joining (right or below). The branches not recovered by one of these analyses are indicated with an asterisk. *fluv*, *L*. *fluviatilis*; *plan*, *L*. *planeri*; am, ammocoete.

The neighbour-joining phylogenetic tree attained from mtDNA sequences is characterized by low structuring, with many basal polytomies (Fig 4). Only three well supported lineages (>80 bootstrap support) were found: two including exclusively haplotypes from the Iberian refugium and a third containing haplotypes from southern France (H10 and H11). The remaining haplotypes representing samples from the Iberian Peninsula and Europe do not show clear genetic structure.

### Putative hybrids between sympatric *L*. *fluviatilis* and *L*. *planeri*

**Fig 5:**
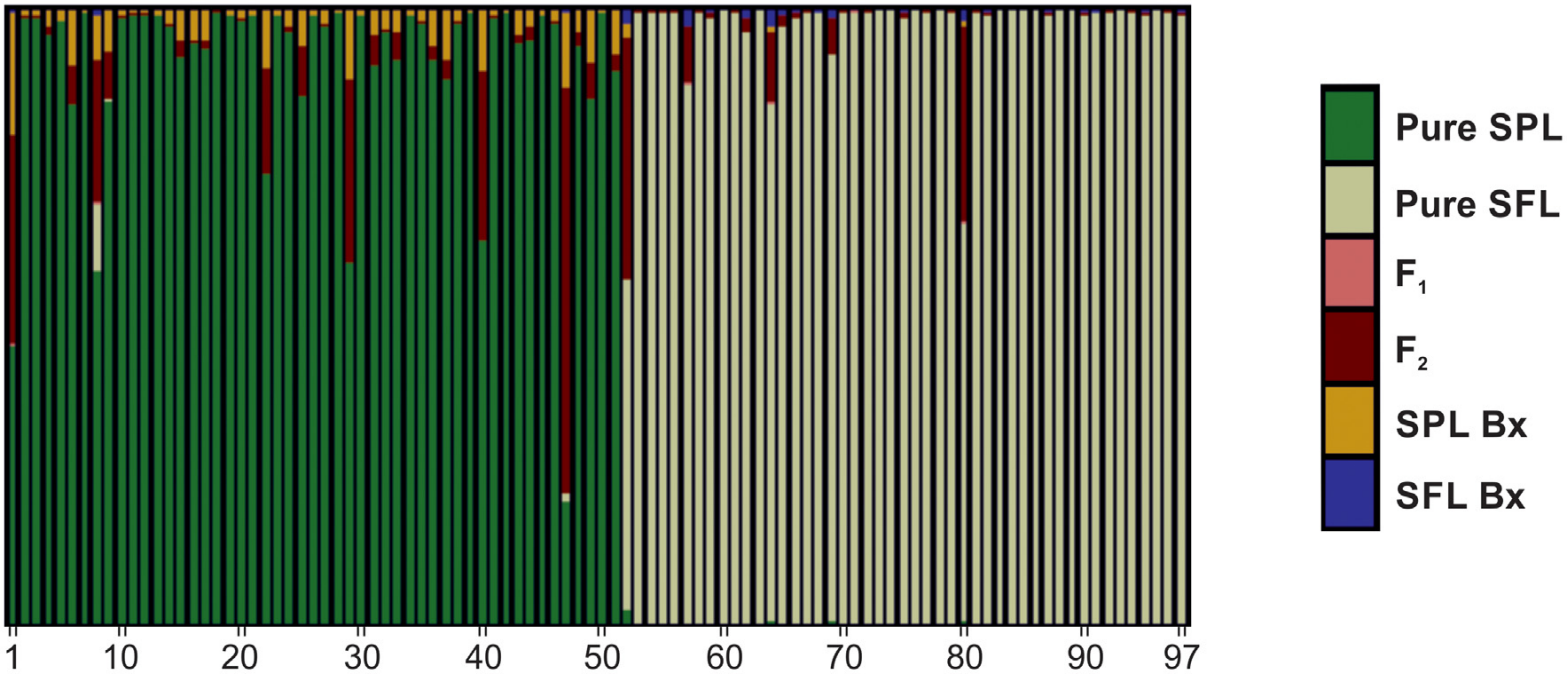
Estimated posterior probabilities that each individual from the sympatric populations of *L*. *planeri* (SPL; *n* = 52) and *L*. *fluviatilis* (SFL; *n* = 46) belongs to each of the six different genotype frequency categories that arise from two generations of potential interbreeding (parental species, F_1_, F_2_ and backcrosses), computed by NewHybrids. Each individual is represented by a vertical bar. For the individuals identified as belonging to a certain hybrid category, posterior probability values are detailed in Table 5.

The existence of putative hybrids between *L*. *planeri* and *L*. *fluviatilis* was investigated using sympatric populations, in this case *L*. *planeri* and *L*. *fluviatilis* from the Tagus basin, Portugal (SPL_*r*_ and SFL_*m*_, respectively), using NewHybrids. In this analysis, each individual was assigned a posterior probability (*p*) of belonging to one of the six different genotype classes resulting from two generations. From the 52 samples of *L*. *planeri*, 49 (94%) were classified as being pure *L*. *planeri* using the posterior probability threshold of 0.5, 20 of which showing *p* > 0.99 and 25 showing 0.8 < *p* < 0.99 (Fig 5). For this species only one individual was classified as hybrid (F_2_; second generation hybrid) with posterior probability of 0.664, and one individual was classified as being pure *L*. *fluviatilis* (posterior probability = 0.537) (Table 5). All the 46 individuals of *L*. *fluviatilis* were identified as such (pure *L*. *fluviatilis*), from which 40 exhibit *p* > 0.99, and 5 showing 0.8 < *p* < 0.99. No F_1_ or backcross hybrids were found in any of the species (Table 5).

**Table 5:** Hybridization analysis for the sympatric *L*. *fluviatilis* (SFL_*m*_) and *L*. *planeri* (SPL_*r*_) from Tagus basin. Estimated posterior probabilities of belonging to one of the six genotype frequency classes (pure parental, F_1_, F_2_ or backcrosses) for the individuals showing some evidence of hybridization. An individual is identified as belonging to a certain class if the posterior probability of falling into that class is above 0.5. Specimens are numbered as in Fig 5.

Species	Specimen	Pure SPL_*r*_	Pure SFL_*m*_	F_1_	F_2_	SPL_*r*_ Bx	SFL_*m*_ Bx
*L*. *planeri* (SPL_*r*_) *n* = 52	1	0.452	-	-	0.342	0.200	-
	8	**0.577**	0.106	-	0.233	0.075	-
	22	**0.733**	-	-	0.171	0.096	-
	29	**0.587**	-	-	0.301	0.113	-
	40	**0.625**	-	-	0.274	0.099	-
	47*	0.198	0.013	-	**0.664**	0.120	-
	52	0.023	**0.537**	-	0.394	0.023	0.022
*L*. *fluviatilis* (SFL_*m*_) *n* = 46	80	-	**0.649**	-	0.319	0.009	0.018

Genotype classes: Pure SPL_*r*_, pure *L*. *planeri*; Pure SFL_*m*_, pure *L*. *fluviatilis*; F_1_, first generation hybrid; F_2_, second generation hybrid; SPL_*r*_ Bx, *L*. *planeri* backcross (pure *L*. *planeri* mating with F_1_); and SFL_*m*_ Bx, *L*. *fluviatilis* backcross (pure *L*. *fluviatilis* mating with F_1_).

Bold indicates the class the individuals were classified into.

*Individual identified as hybrid.

### Migration rate among populations

**Fig 6:**
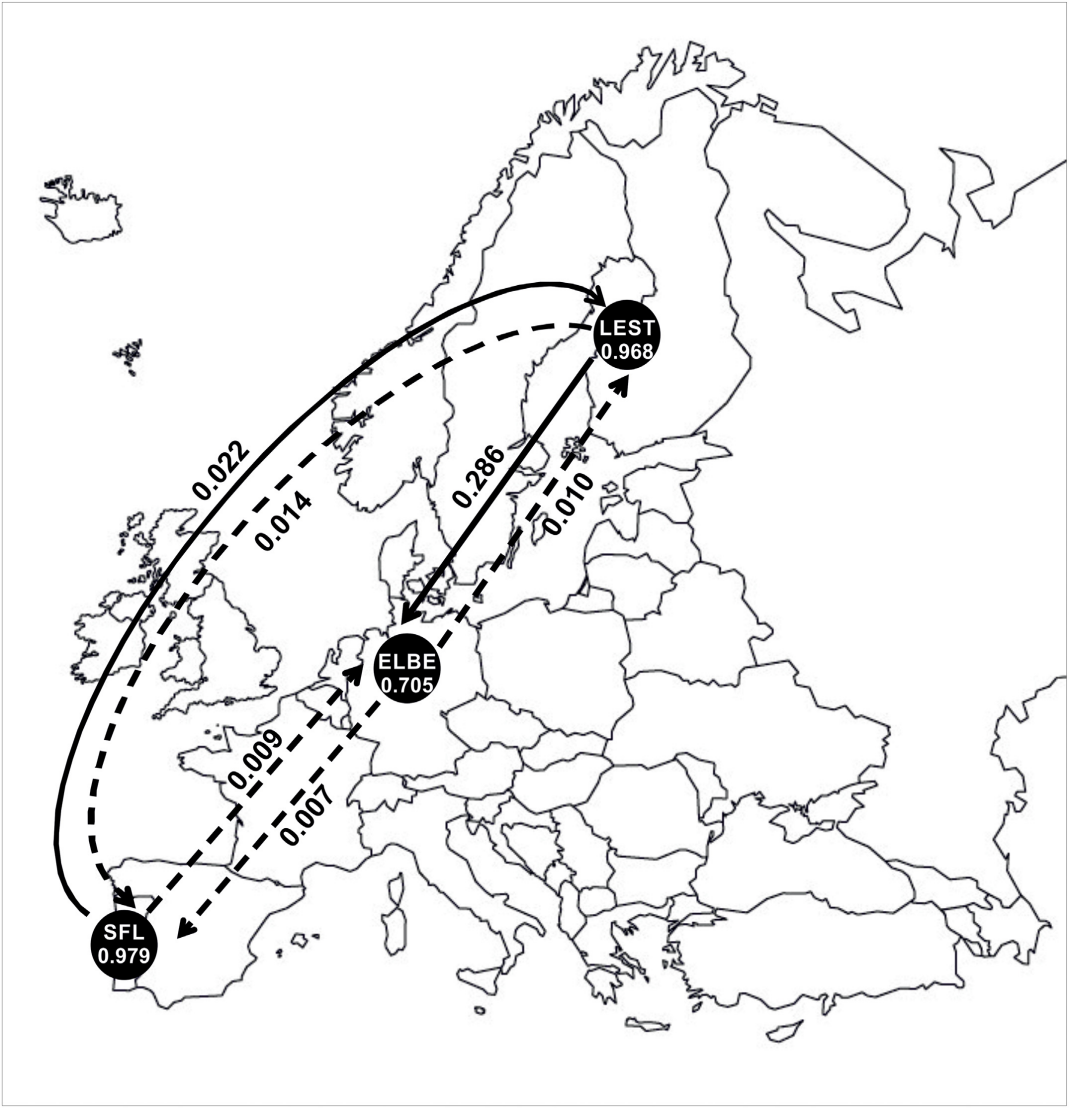
Recent migration rates (*m*) between migratory populations estimated using BAYESASS. Within circles, acronyms represent samples as in Fig 1 and Table 1, and numbers denote the proportion of non-immigrants within populations. Arrows indicate direction of gene flow among populations and respective *m* value. Dashed arrows represent values of *m* lower than 0.02.

Recent migration rates (*m*) among migratory populations were estimated using BAYESASS. This analysis was performed for the three anadromous populations included in the study, i.e., *L*. *fluviatilis* from Tagus (SFL_*m*_), from Elbe (ELBE_*m*_) and from Lestijoki (LEST_*m*_). The proportion of individuals derived from their own location was high in SFL_*m*_ (*m* = 0.979) and in LEST_*m*_ (*m* = 0.968), and relatively low in ELBE_*m*_ (*m* = 0.705) (Fig 6). Accordingly, a relatively high proportion of immigrants (*m* = 0.286) was detected from LEST_*m*_ into ELBE_*m*_. SFL_*m*_ is the most isolated population, with the highest proportion of non-immigrants (*m* = 0.979) and low migration rates (*m*≤0.02) in both directions (Fig 6, Table 6).

**Table 6:** Bayesian estimates of recent migration rates (*m*) among all populations using the program BAYESASS. Values shown are the mean migration rate into each population and their respective standard deviation of the marginal posterior distribution in parentheses. Values along the diagonal (in bold) represent the proportion of non-immigrants within populations. Values of *m* higher than 0.02 are presented in italics.

	Migration into										
	LEST_*m*_	BEKE_*r*_	ELBE_*m*_	WARC_*r*_	ESM_*r*_	LIS_*r*_	OES_*r*_	NAB_*r*_	SPL_*r*_	SFL_*m*_	SADO_*r*_
Migration from											
LEST_*m*_	**0.9019 (0.0274)**	*0*.*2554* (0.0216)	*0*.*2428* (0.0205)	0.0074 (0.0071)	0.0079 (0.0075)	0.0071 (0.0070)	0.0073 (0.0071)	0.0071 (0.0068)	0.0067 (0.0063)	0.0141 (0.0112)	0.0053 (0.0053)
BEKE_*r*_	0.0083 (0.0082)	**0.6753 (0.0084)**	0.0064 (0.0063)	0.0072 (0.0070)	0.0076 (0.0075)	0.0073 (0.0071)	0.0073 (0.0071)	0.0072 (0.0070)	0.0057 (0.0056)	0.0059 (0.0058)	0.0054 (0.0054)
ELBE_*m*_	0.0085 (0.0082)	0.0076 (0.0074)	**0.6995 (0.0136)**	0.0071 (0.0070)	0.0075 (0.0074)	0.0072 (0.0070)	0.0076 (0.0073)	0.0073 (0.0070)	0.0061 (0.0059)	0.0060 (0.0058)	0.0053 (0.0052)
WARC_*r*_	0.0082 (0.0079)	0.0077 (0.0075)	0.0063 (0.0062)	**0.9261 (0.0210)**	0.0074 (0.0071)	0.0073 (0.0069)	0.0076 (0.0077)	0.0115 (0.0094)	0.0057 (0.0057)	0.0060 (0.0058)	0.0054 (0.0053)
ESM_*r*_	0.0085 (0.0083)	0.0077 (0.0075)	0.0061 (0.0060)	0.0076 (0.0075)	**0.9244 (0.0209)**	0.0071 (0.0069)	0.0075 (0.0074)	0.0076 (0.0074)	0.0187 (0.0131)	0.0058 (0.0058)	0.0054 (0.0052)
LIS_*r*_	0.0084 (0.0082)	0.0077 (0.0073)	0.0062 (0.0059)	0.0071 (0.0070)	0.0074 (0.0072)	**0.6745 (0.0076)**	*0*.*2579* (0.0210)	0.0071 (0.0071)	0.0053 (0.0052)	0.0058 (0.0057)	0.0054 (0.0053)
OES_*r*_	0.0083 (0.0082)	0.0077 (0.0075)	0.0065 (0.0063)	0.0070 (0.0070)	0.0075 (0.0077)	*0*.*2609* (0.0196)	**0.6747 (0.0078)**	0.0074(0.0075)	0.0052 (0.0052)	0.0059 (0.0059)	0.0056 (0.0055)
NAB_*r*_	0.0085 (0.0082)	0.0078 (0.0077)	0.0061 (0.0061)	0.0073 (0.0070)	0.0075 (0.0073)	0.0072 (0.0068)	0.0076 (0.0075)	**0.9228 (0.0208)**	0.0055 (0.0055)	0.0059 (0.0058)	0.0053 (0.0053)
SPL_*r*_	0.0081 (0.0081)	0.0078 (0.0075)	0.0063 (0.0059)	0.0076 (0.0076)	0.0076 (0.0073)	0.0073 (0.0070)	0.0073 (0.0071)	0.0073 (0.0072)	**0.9272 (0.0195)**	0.0059 (0.0057)	0.0053 (0.0053)
SFL_*m*_	*0*.*0229* (0.0189)	0.0076 (0.0075)	0.0076 (0.0072)	0.0083 (0.0082)	0.0075 (0.0073)	0.0071 (0.0069)	0.0076 (0.0074)	0.0074 (0.0072)	0.0083 (0.0074)	**0.9330 (0.0191)**	0.0055 (0.0055)
SADO_*r*_	0.0084 (0.0081)	0.0076 (0.0075)	0.0063 (0.0063)	0.0074 (0.0074)	0.0076 (0.0073)	0.0071 (0.0071)	0.0075 (0.0073)	0.0073 (0.0072)	0.0054 (0.0053)	0.0059 (0.0057)	**0.9462 (0.0154)**

### Demographic history

Bottleneck analysis revealed consistent signs for recent contraction of population size only in the population from Meuse (WARC_*r*_), which showed significant (*P*<0.05) heterozygote excess according to the three mutational models tested, and a shift in the distribution of allele frequency classes from the expected L-shaped distribution (Table 7). The population from Nabão (NAB_*r*_) also presents significant (*P*<0.01) heterozygote excess according to IAM, but no deviation from the expected L-shaped distribution (Table 7). Using the *M* ratio statistic test, we found strong evidence of past reduction in population size for the migratory *L*. *fluviatilis* from Tagus (SFL_*m*_), as the *M* ratio was significantly smaller than the equilibrium expectation (*P*<0.05) for all the prebottleneck *θ* values considered. None of the remaining populations presented signs of reduction in population size (Table 7).

**Table 7:** Demographic analysis. ***P* values for one-tailed heterozygote excess (bold indicates significant *P* values), deviation of allele frequency classes from a normal L-shaped distribution and *M* ratio tests. For populations’ acronyms, please check Table 1**.

	IAM	TPM	SMM	L-shape	*M* ratio value
LEST_*m*_	0.326	0.820	0.993	no deviation	0.72
BEKE_*r*_	0.578	0.963	0.994	no deviation	0.75
ELBE_*m*_	0.248	0.590	0.936	no deviation	0.71
WARC_*r*_	**0.014**	**0.020**	**0.037**	deviation	0.79
ESM_*r*_	0.156	0.563	0.906	no deviation	0.83
NAB_*r*_	**0.007**	0.064	0.082	no deviation	0.75
SPL_*r*_	0.097	0.216	0.784	no deviation	0.78
SFL_*m*_	0.326	0.674	0.976	no deviation	0.66^1^
SADO_*r*_	0.422	0.422	0.578	no deviation	0.82

IAM, infinite allele model; TPM, two-phase model; SMM, stepwise mutation model.

The populations LIS_*r*_ and OES_*r*_ were not included in the analysis as they only have one polymorphic locus (Lspn 094) (S2 Table).

^1^Location with *M* ratio value significantly smaller than the equilibrium expectation (*P* < 0.05) for all the prebottleneck θ values considered (4, 10 and 20).

## Discussion

### Genetic diversity, population structure and postglacial dispersal

The colonization processes that took place after the glacial periods, when populations from the southern Mediterranean peninsulas expanded north across Europe, shaped, together with recent processes, the biodiversity of current taxa. Southern populations isolated in refugia and sub-refugia accumulated variation through the ice ages, and the founders that rapidly moved northward during interglacials only represented a subsample of the southern diversity [46]. This pattern is clearly observed for the genus *Lampetra* in this study. This genus presents a higher number of species and higher genetic diversity in the Iberian Peninsula compared with those of central and northern Europe, with the majority of private alleles being found in southern samples. This pattern of reduced richness in northern regions has already been observed by Bracken *et al*. [47] for the same species inhabiting the British Isles, and by Boguski *et al*. [48] for North American *Lampetra*, where haplotypic richness is greatest in regions south of the Columbia River. Similarly, Goodman *et al*. [49] and Spice *et al*. [50] found that in the anadromous Pacific lamprey (*Entosphenus tridentatus*), haplotypic richness increases from north to south.

This study is another evidence of the Iberian Peninsula as a glacial refugium, as proposed by Taberlet *et al*. [1], and posteriorly supported by several case-studies, namely in fish. For instance, a number of studies on endemic Iberian cyprinid species support this scenario, like for *Barbus sclateri* [51], *Squalius aradensis* [52] and *Pseudochondrostoma polylepis* [53]. Likewise, the study of Consuegra *et al*. [54] with Atlantic salmon demonstrates the presence of this species in the Iberian glacial refugium during the last 40 000 years and also points to the Iberian Peninsula as the likely source of the most common haplotype within the Atlantic lineage in Europe.

STRUCTURE analysis for the 11 analyzed samples revealed the existence of eight genetic clusters: whereas the southern populations were grouped in several genetic clusters reflecting their high levels of differentiation, northern populations were grouped in the same genetic cluster as result of their more recent common ancestor. The exception was the resident *L*. *planeri* from Warche in Belgium, which constitutes a distinct genetic cluster with high proportion of membership. This population seems to be facing a genetic bottleneck (see Table 6), which explains the relatively low number of alleles found in each microsatellite locus (maximum of three, see S2 Table). This may reflect the isolation of this population, which is located very upstream in the river basin, with several important obstacles downstream (M. Ovidio pers. comm.). Glaciations have been considered an important factor in brook lamprey evolution, both in Europe and North America [48,55,56]. These long periods favoured the abandonment of anadromous habits due to blocking of migratory routes. A cryptic Belgian refugium is another hypothesis for this differentiation, as several cryptic northern refugia have been hypothesized, one of which in the Belgian Ardennes. These northern refugia would have been in areas of sheltered topography that provided suitable stable microclimates [57]. We tested, using mtDNA, whether the population from Warche represent an independently evolved population that survived during glaciations in the Belgian Ardennes refugium (see Fig 4). The results attained were not conclusive because although the three private haplotypes from Warche (H83 to H85) group in a clade, this branch shows low support.

Demographic analyses revealed consistent signs for recent contraction of population size in the population from Meuse (WARC_*r*_). Nabão (NAB_*r*_) population presented significant heterozygote excess according to the infinite allele model (IAM). These results, however, were not corroborated by mtDNA data, where Fu’s *Fs* [58] and Tajima’s *D* [59] statistics revealed non-significant values (WARC_*r*_ calculated in the present study, and NAB_*r*_ from Mateus *et al*. [9]), unlike what would be expected from a recent population bottleneck. This might be explained by the different signals attained from both markers, i.e., the more ancestral signal retrieved from mtDNA *versus* the recent history retrieved from microsatellite loci. This would mean that populations historically not showing evidence for contraction in population size (as attained from mtDNA data), may have faced a recent genetic bottleneck, as revealed by microsatellite data. Grouping of Iberian populations by STRUCTURE reflects in general their specific status, with the exception of LIS_*r*_ and OES_*r*_ that were grouped together, but not grouped with the other population of *L*. *planeri* from Portugal (SPL_*r*_). Those two populations present very low levels of genetic diversity, having one single polymorphic locus and no private alleles. In general, the alleles present in those populations are rather common, most of the times having a frequency of more than 50% in other populations, namely SPL_*r*_. The differentiation of those populations from SPL_*r*_ seems, therefore, to reflect this lack of diversity, which statistically makes them unique. Those populations may be facing a genetic bottleneck, but this analysis could not be performed due to the existence of a single polymorphic locus.

This study revealed that anadromous populations from central and northern Europe have high proportions of membership from the population of *L*. *fluviatilis* from the Iberian Peninsula (the SFL_*m*_ genetic cluster). Because contemporary gene flow from south to north and vice-versa is apparently happening in very small proportions (see Fig 6), this signal is likely due to ancestral polymorphism, as a result of the colonization process from a southern *L*. *fluviatilis*-type, rather than ongoing gene flow. The hypothesis of ancestral polymorphism is also supported by the mtDNA analysis, as northern samples of *L*. *fluviatilis* (LEST_*m*_ and ELBE_*m*_) share haplotype 62 with *L*. *fluviatilis* from the Tagus watershed (Iberian Peninsula) (see Fig 4), and is in tune with the scenario of recent dispersal and founding of the northern populations ([9,11]; discussed above). For a better understanding of the contemporary patterns of gene flow in more recently established northern populations, recent migration routes among and within a wider range of northern *L*. *fluviatilis* and *L*. *planeri* populations should be further investigated.

### Gene flow among species and populations

The repeated emergence of resident forms from ancestral migratory ones in different locations and times is a phenomenon very well known in lampreys [5–7,60], and should promote varying degrees of reproductive isolation between founder and derived species.

Previous data on mtDNA suggested that the three Iberian brook lampreys *L*. *alavariensis*, *L*. *auremensis* and *L*. *lusitanica* are well supported species, while the brook *L*. *planeri* shares haplotypes with the parasitic form, which is evidence that its emergence was more recent [9,11]. Present microsatellite data is concordant with the mtDNA data, as it revealed no signal of ongoing gene flow between the Iberian endemics with any of the samples included in this study. In the paired *L*. *planeri* and *L*. *fluviatilis* the scenario is different, as levels of genetic differentiation vary among populations. Sympatric *L*. *fluviatilis* and *L*. *planeri* from the Tagus basin present significant genetic differentiation and almost no signal of hybridization. These results are in agreement with the previous work using restriction site-associated DNA (RAD) sequencing [14] that suggests that these populations are two distinct taxa that diverged recently. Results attained for northern populations of *L*. *fluviatilis* and *L*. *planeri*, however, indicate that these species are grouped in a single cluster (STRUCTURE analysis) and may be experiencing, or have experienced until recently, gene flow. This scenario is most likely explained by the postglacial colonization of northern habitats by a southern *L*. *fluviatilis*-type and, consequently, later emergence of northern populations, as explained below. Differentiation between resident and anadromous populations may, therefore, be an ongoing process in many locations, where speciation is still underway. This scenario was also found for the same species in France, where variable levels of divergence were found among sympatric population pairs [61]. This may also be the case in other lamprey paired species; for instance, no significant genetic differences were found between sympatric populations of the paired silver (*Ichthyomyzon unicuspis*) and northern brook (*Ichthyomyzon fossor*) lampreys from the Great Lakes, suggesting the existence of ongoing gene flow between them at least in this region [62].

BAYESASS revealed high recent gene flow between the migratory northern populations ELBE_*m*_ and LEST_*m*_, which is corroborated by the assignment tests (see Table 4). In contrast, the migratory *L*. *fluviatilis* from the Iberian Peninsula has a signature similar to a resident species, showing almost absence of ongoing gene flow with northern populations and high degree of isolation and differentiation. This is consistent with the findings of [63], who suggested that lampreys with smaller body size may show limited dispersal and greater genetic differentiation. The genetic isolation of *L*. *fluviatilis* from the Iberian Peninsula, together with the relatively small size of individuals may reveal reduced levels of mobility during the parasitic adult phase, probably associated with their permanence in the large Tagus estuary (ca. 300 km^2^) and adjacent coastal area. *Lampetra fluviatilis* migrants have been separated on the basis of size into “typical” and “praecox” forms, whose mean lengths are approximately 30 and 24 cm, and mean weights about 53 and 22 g, respectively [64]. The size difference between the typical and praecox forms is thought to be due to differences in the time spent feeding in the sea, the last reducing their marine feeding phase by at least 1 year [64]. The population from the Tagus river basin resembles these smaller praecox forms; specimens analysed by the authors had in average 26 cm total length and 33 g weight, and one of the individuals was as small as 20 cm of total length and 19 g weight. These low values contrast, for instance, with those registered by Kemp *et al*. [65] for this species in north-east England, 80.7 g and 36.3 cm. The southern population from Tagus river basin shows strong evidence of past reduction in population size (this study), and the low number of individuals caught in the last years is representative of the rareness of this population [10], which is apparently isolated from the remaining European populations.

## Conclusions

The use of microsatellites, coupled with information of mtDNA data, has proved effective in unravelling the patterns of colonization in European *Lampetra*, placing the Iberian Peninsula as a major source of postglacial colonization for this genus. We suggest the existence of a speciation continuum between *L*. *fluviatilis* and *L*. *planeri*, i.e., different populations of this pair likely represent differentiation at different stages, as result of the different timing of colonization and refugial persistence. This hypothesis should be further investigated with the addition of more samples, namely of sympatric populations.

Gene flow between migratory populations is high in northern regions, but low between northern populations and the southern Iberian population. We hypothesise that the migratory *L*. *fluviatilis* from the Iberian Peninsula has limited dispersion movements, resembling the “praecox” form described for this species by Abou-Seedo and Potter (1979).

## Supporting Information

S1 Fig Analysis of convergence of the MCMC algorithm for the estimates from BAYESASS (Bayesian estimates of recent migration rates) using the software Tracer 1.6. Plots represent the log probability along iterations, where the burn-in iterations are indicated in light grey and sample iterations in black; and the Bayesian posterior density of the parameter estimates for a) the three migratory populations and b) all the 11 populations. As should be expected in a case of convergence, the log probability oscillates around a plateau, and the oscillations are quite regular, i.e., there are no persistent lows or highs (valleys or hills) in the plot.
(DOCX)

S1 Table List of the 10 polymorphic primer sets used, allelic range (bp), fluorescent label and multiplex panel. (DOCX)

S2 Table Measures of genetic diversity assayed at ten microsatellite DNA loci for each sampled location. Sample acronyms correspond to locations as in Fig 1 and Table 1. Number of alleles per locus (Na) with number of private alleles in parentheses, mean allelic richness (AR), unbiased expected heterozygosity (He), observed heterozygosity (Ho), significance of departure from Hardy–Weinberg Equilibrium (HWE), mean number of alleles across loci (MNA) and number of polymorphic loci in each location (P). Grey shading indicates loci where MICRO-CHECKER detected signs of null alleles and relation with deviations from Hardy–Weinberg equilibrium. NS, non-significant; *, *P*<0.05; **, *P*<0.01; ***, *P*<0.001; *n*, sample size; †, private allele with frequency >50%.
(DOCX)

S3 Table Data of the ten microsatellite loci assayed for each of the 415 samples. (PDF)
