# Supplementary material for: European Lampreys: New Insights on Postglacial Colonization, Gene Flow and Speciation
Source: PLoS One. 2016 Feb 12;11(2):e0148107. doi: 10.1371/journal.pone.0148107 (PMC4752455; doi:10.1371/journal.pone.0148107)
Supplement: S1 Fig — Plots represent the log probability along iterations, where the burn-in iterations are indicated in light grey and sample iterations in black; and the Bayesian posterior density of the parameter estimates for a) the three migratory populations and b) all the 11 populations. As should be expected in a case of convergence, the log probability oscillates around a plateau, and the oscillations are quite regular, i.e., there are no persistent lows or highs (valleys or hills) in the plot. (DOCX) [file pone.0148107.s001.docx]

**S1 Fig.** Analysis of convergence of the MCMC algorithm for the estimates from BAYESASS (Bayesian estimates of recent migration rates) using the software Tracer 1.6 [1].

Plots represent the log probability along iterations, where the burn-in iterations are indicated in light grey and sample iterations in black; and the Bayesian posterior density of the parameter estimates for a) the three migratory populations and b) all the 11 populations.

As should be expected in a case of convergence, the log probability oscillates around a plateau, and the oscillations are quite regular, i.e., there are no persistent lows or highs (valleys or hills) in the plot.

| **a)**  **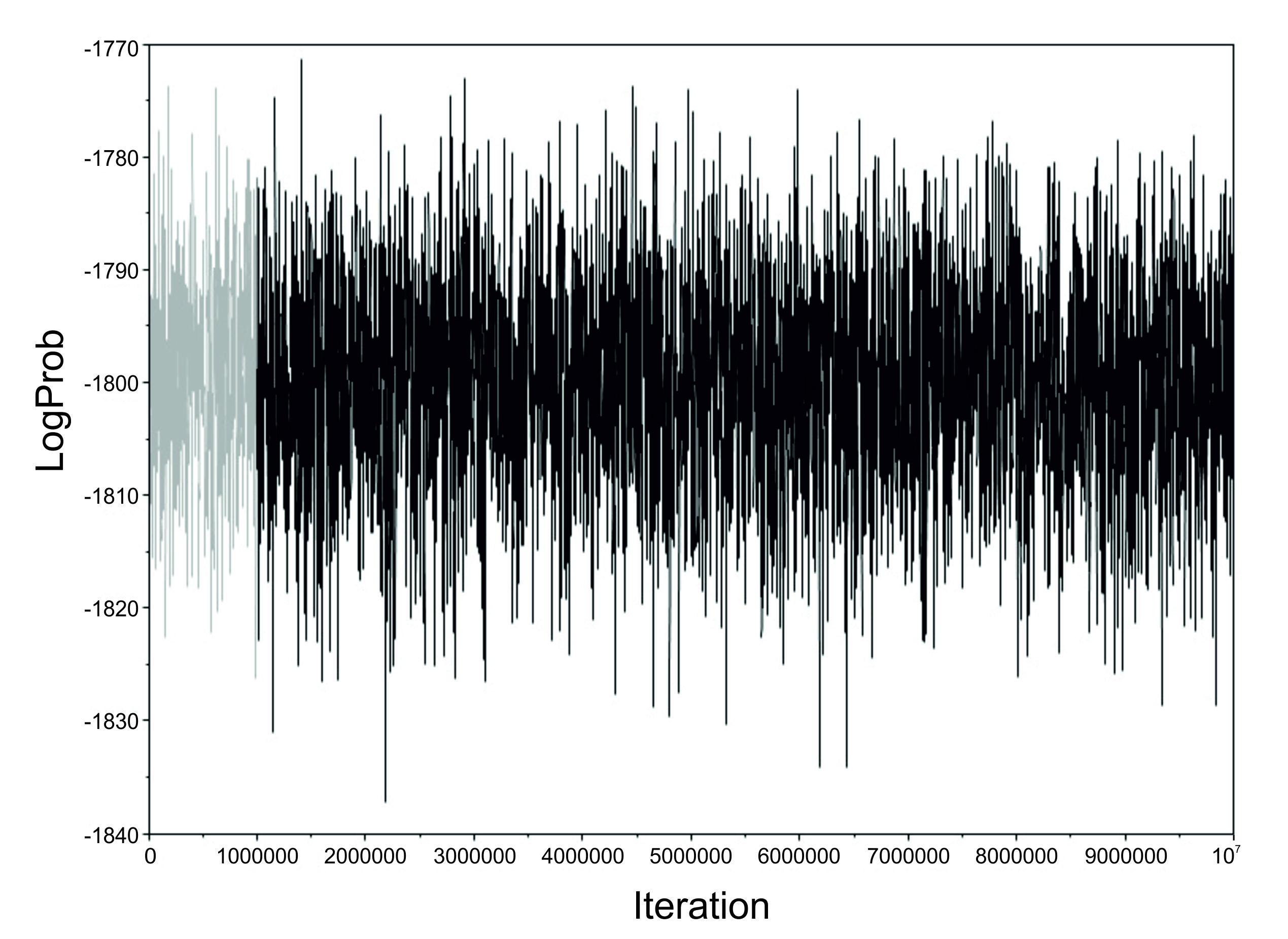**  **b)** | **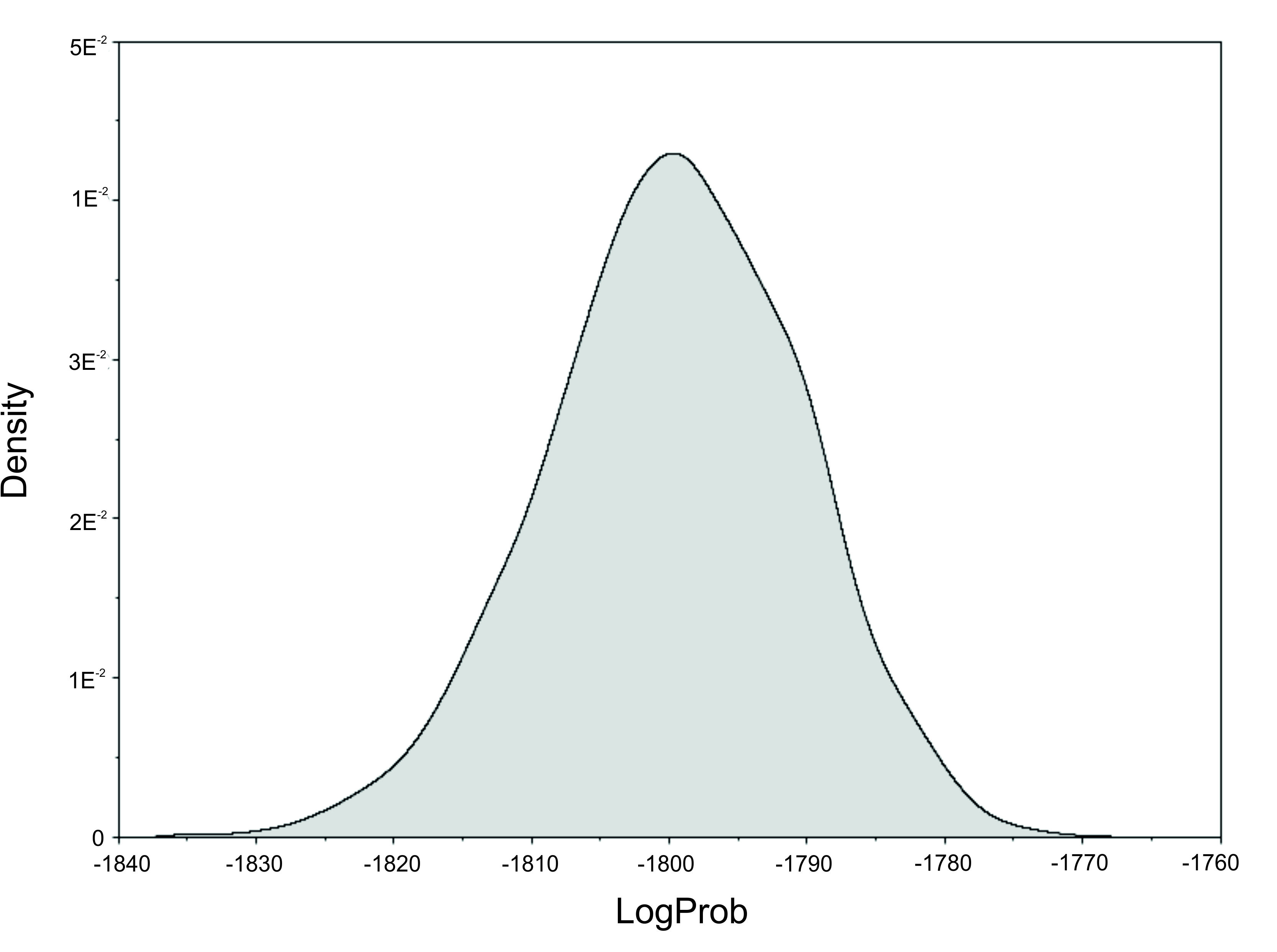** |
| --- | --- |
| **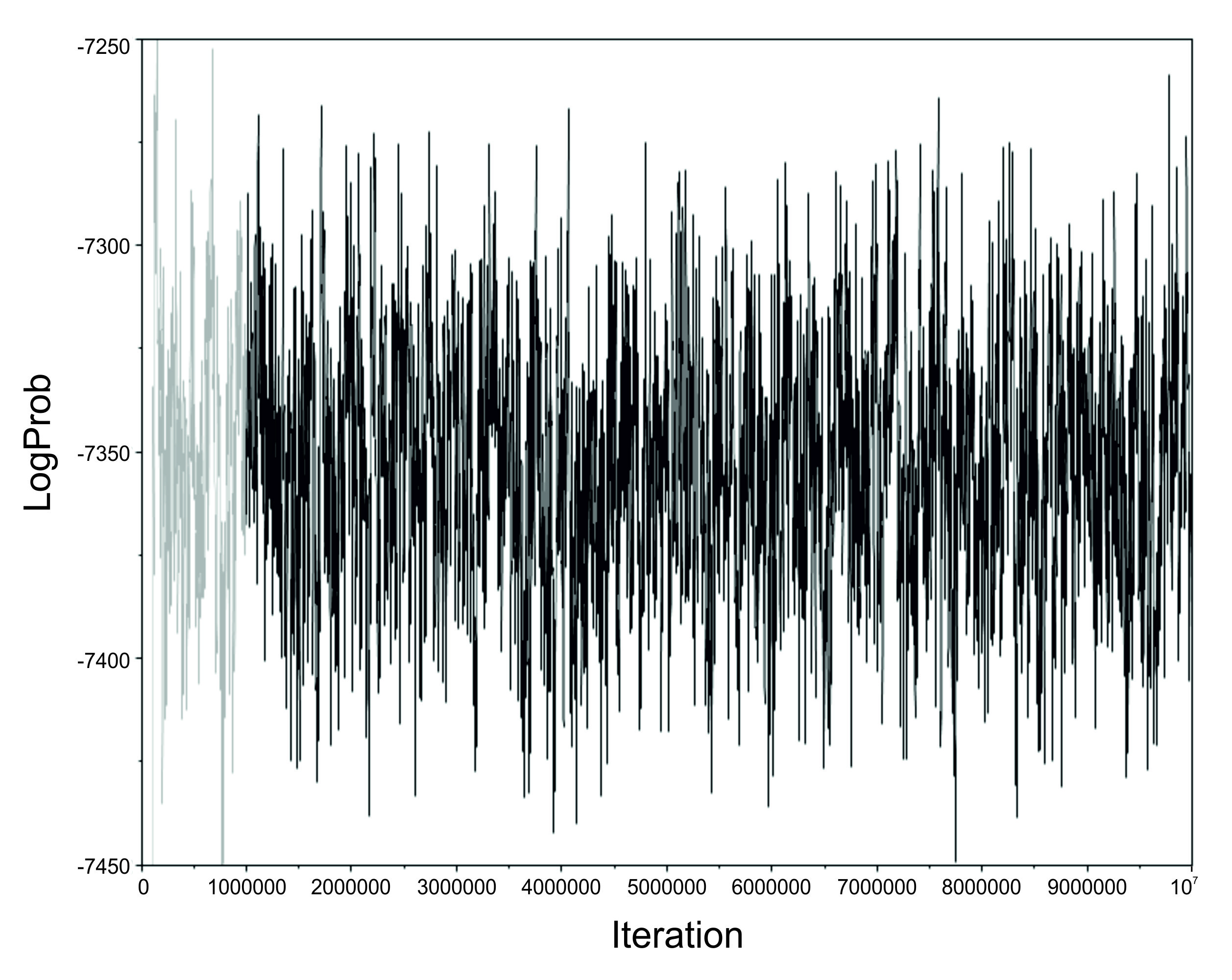** | **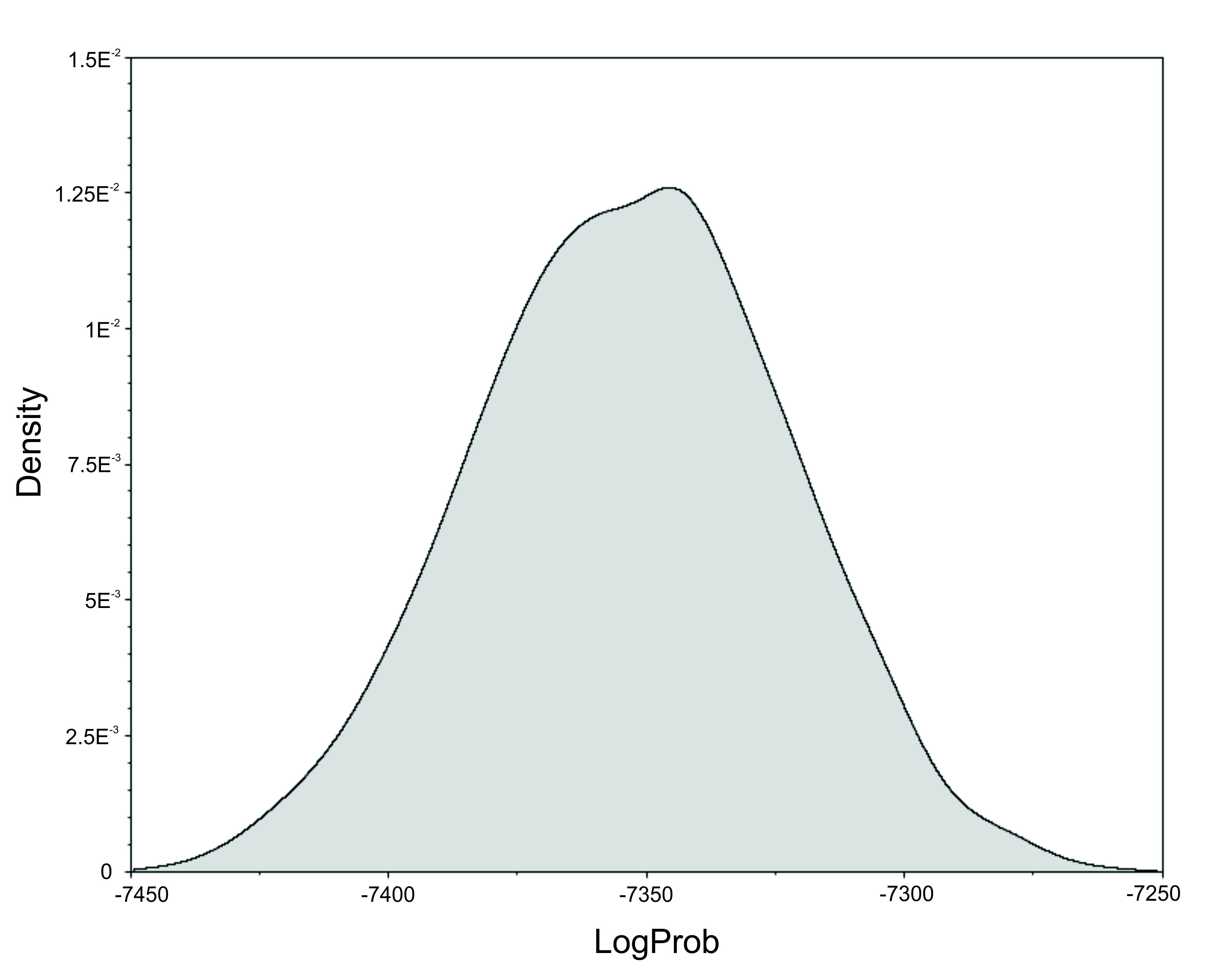** |

**References**

1. Rambaut A, Suchard MA, Xie D, Drummond AJ (2014) Tracer v1.6, Available from http://beast.bio.ed.ac.uk/Tracer.
