# Supplementary material for: European Lampreys: New Insights on Postglacial Colonization, Gene Flow and Speciation
Source: PLoS One. 2016 Feb 12;11(2):e0148107. doi: 10.1371/journal.pone.0148107 (PMC4752455; doi:10.1371/journal.pone.0148107)
Supplement: S1 Table — (DOCX) [file pone.0148107.s002.docx]

**S1 Table. List of the 10 polymorphic primer sets used, allelic range (bp), fluorescent label and multiplex panel.**

| **Locus** | **Allele size range (bp)** | **Fluorescent label/ multiplex panel** |
| --- | --- | --- |
| Iun 2 | 120-129 | PET/1 |
| Iun 5 | 246-312 | 6-FAM/2 |
| Iun 7 | 179-181 | VIC/1 |
| Iun 10 | 125-191 | PET/2 |
| Iun 14 | 369-451 | NED/1 |
| Lspn 010-2 | 204-208 | NED/1 |
| Lspn 019c | 136-146 | VIC/3 |
| Lspn 044 | 196-216 | 6-FAM/3 |
| Lspn 094 | 180-208 | NED/2 |
| Pma*μ* 5 | 241-251 | VIC/3 |
