# Supplementary material for: European Lampreys: New Insights on Postglacial Colonization, Gene Flow and Speciation
Source: PLoS One. 2016 Feb 12;11(2):e0148107. doi: 10.1371/journal.pone.0148107 (PMC4752455; doi:10.1371/journal.pone.0148107)
Supplement: S2 Table — Sample acronyms correspond to locations as in Fig 1 and Table 1. Number of alleles per locus (Na) with number of private alleles in parentheses, mean allelic richness (AR), unbiased expected heterozygosity (He), observed heterozygosity (Ho), significance of departure from Hardy–Weinberg Equilibrium (HWE), mean number of alleles across loci (MNA) and number of polymorphic loci in each location (P). Grey shading indicates loci where MICRO-CHECKER detected signs of null alleles and relation with deviations from Hardy–Weinberg equilibrium. NS, non-significant; *, P<0.05; **, P<0.01; ***, P<0.001; n, sample size; †, private allele with frequency >50%. (DOCX) [file pone.0148107.s003.docx]

S2 Table. Measures of genetic diversity assayed at ten microsatellite DNA loci for each sampled location. Sample acronyms correspond to locations as in Fig. 1 and Table 1. Number of alleles per locus (Na) with number of private alleles in parentheses, mean allelic richness (AR), unbiased expected heterozygosity (He), observed heterozygosity (Ho), significance of departure from Hardy–Weinberg Equilibrium (HWE), mean number of alleles across loci (MNA) and number of polymorphic loci in each location (P). Grey shading indicates loci where MICRO-CHECKER 2.2.3 [1] detected signs of null alleles and relation with deviations from Hardy–Weinberg equilibrium. NS, non-significant; *, *P*<0.05; **, *P*<0.01; ***, *P*<0.001; *n*, sample size; †, private allele with frequency >50%.

| Sample | LEST*_m_* | BEKE*_r_* | ELBE*_m_* | WARC*_r_* | ESM*_r_* | LIS*_r_* | OES*_r_* | NAB*_r_* | SPL*_r_* | SFL*_m_* | SADO*_r_* | Overall |
| --- | --- | --- | --- | --- | --- | --- | --- | --- | --- | --- | --- | --- |
|  | *n*=29 | *n*=30 | *n*=40 | *n*=35 | *n*=33 | *n*=33 | *n*=31 | *n*=35 | *n*=52 | *n*=46 | *n*=51 |  |
| *Locus* |  |  |  |  |  |  |  |  |  |  |  |  |
| **Iun 2** |  |  |  |  |  |  |  |  |  |  |  |  |
| Allelic range (bp) | 123-129 | 123-129 | 123-129 | 123-126 | 123 | 123 | 123 | 120-123 | 120-126 | 123-126 | 123-126 | 120-129 |
| Na | 3 | 3 | 3 | 2 | 1 | 1 | 1 | 2 | 3 | 2 | 2 | 4 |
| AR | 1.9755 | 2.0493 | 1.8841 | 1.9397 | 1.0000 | 1.0000 | 1.0000 | 1.9978 | 2.8435 | 1.5660 | 1.4096 |  |
| He | 0.2716 | 0.2672 | 0.2434 | 0.3578 | 0.0000 | 0.0000 | 0.0000 | 0.4969 | 0.5937 | 0.1421 | 0.0942 |  |
| Ho | 0.3103 | 0.3000 | 0.2750 | 0.4000 | 0.0000 | 0.0000 | 0.0000 | 0.5143 | 0.4600 | 0.1522 | 0.0588 |  |
| HWE | NS | NS | NS | NS | - | - | - | NS | NS | NS | NS |  |
| **Iun 5** |  |  |  |  |  |  |  |  |  |  |  |  |
| Allelic range (bp) | 246-267 | 246-312 | 246-297 | 246-258 | 255-282 | 252 | 252 | 252 | 252-258 | 252-282 | 249-252 | 246-312 |
| Na | 5 | 6 (1) | 7 | 3 | 2 | 1 | 1 | 1 | 3 | 4 | 2 (1†) | 9 |
| AR | 3.0616 | 3.7347 | 3.8711 | 2.3257 | 1.2821 | 1.0000 | 1.0000 | 1.0000 | 1.8593 | 2.2939 | 1.9978 |  |
| He | 0.5793 | 0.6836 | 0.6452 | 0.4352 | 0.0597 | 0.0000 | 0.0000 | 0.0000 | 0.2417 | 0.2871 | 0.4980 |  |
| Ho | 0.5556 | 0.3793 | 0.4545 | 0.3429 | 0.0606 | 0.0000 | 0.0000 | 0.0000 | 0.2745 | 0.2273 | 0.6078 |  |
| HWE | NS | *** | *** | NS | NS | - | - | - | NS | NS | NS |  |
| **Iun 7** |  |  |  |  |  |  |  |  |  |  |  |  |
| Allelic range (bp) | 179-181 | 179 | 179-181 | 179-181 | 179 | 179 | 179 | 179-181 | 179-181 | 179-181 | 179 | 179-181 |
| Na | 2 | 1 | 2 | 2 | 1 | 1 | 1 | 2 | 2 | 2 | 1 | 2 |
| AR | 1.5414 | 1.0000 | 1.8503 | 1.9937 | 1.0000 | 1.0000 | 1.0000 | 1.8416 | 1.9975 | 1.8255 | 1.0000 |  |
| He | 0.1307 | 0.0000 | 0.2755 | 0.4737 | 0.0000 | 0.0000 | 0.0000 | 0.2687 | 0.4957 | 0.2609 | 0.0000 |  |
| Ho | 0.1379 | 0.0000 | 0.2162 | 0.6286 | 0.0000 | 0.0000 | 0.0000 | 0.3143 | 0.7885 | 0.2609 | 0.0000 |  |
| HWE | NS | - | NS | NS | - | - | - | NS | *** | NS | - |  |
| **Iun 10** |  |  |  |  |  |  |  |  |  |  |  |  |
| Allelic range (bp) | 137-188 | 179-185 | 137-191 | 137-191 | 185 | 188 | 188 | 182-185 | 173-191 | 137-188 | 125-188 | 125-191 |
| Na | 5 | 3 | 6 | 3 | 1 | 1 | 1 | 2 | 6 (1) | 5 | 5 (1†) | 8 |
| AR | 3.6206 | 2.2174 | 4.0753 | 2.8050 | 1.0000 | 1.0000 | 1.0000 | 1.9986 | 4.1921 | 3.3064 | 1.8967 |  |
| He | 0.6836 | 0.3181 | 0.7034 | 0.6195 | 0.0000 | 0.0000 | 0.0000 | 0.5035 | 0.7633 | 0.6467 | 0.1888 |  |
| Ho | 0.6207 | 0.1000 | 0.6389 | 0.5143 | 0.0000 | 0.0000 | 0.0000 | 0.3824 | 0.3137 | 0.5870 | 0.1429 |  |
| HWE | NS | *** | NS | NS | - | - | - | NS | *** | NS | * |  |
| **Iun 14** |  |  |  |  |  |  |  |  |  |  |  |  |
| Allelic range (bp) | 371-425 | 373-425 | 371-425 | 395 | 395-425 | 379 | 379 | 375-415 | 369-425 | 371-425 | 373-451 | 369-451 |
| Na | 6 | 5 | 7 | 1 | 3 | 1 | 1 | 4 | 7 (2) | 5 | 2 (1) | 13 |
| AR | 3.8329 | 3.5045 | 3.4953 | 1.0000 | 2.1686 | 1.0000 | 1.0000 | 3.0652 | 3.3168 | 3.4413 | 1.4096 |  |
| He | 0.6370 | 0.6763 | 0.5642 | 0.0000 | 0.3506 | 0.0000 | 0.0000 | 0.6220 | 0.6306 | 0.6276 | 0.0942 |  |
| Ho | 0.7586 | 0.4667 | 0.5500 | 0.0000 | 0.3030 | 0.0000 | 0.0000 | 0.6765 | 0.4118 | 0.5870 | 0.0980 |  |
| HWE | NS | * | NS | - | NS | - | - | NS | ** | NS | NS |  |
| **Lspn 010-2** |  |  |  |  |  |  |  |  |  |  |  |  |
| Allelic range (bp) | 208 | 208 | 208 | 208 | 208 | 208 | 208 | 204-208 | 204-208 | 208 | 208 | 204-208 |
| Na | 1 | 1 | 1 | 1 | 1 | 1 | 1 | 2 | 2 | 1 | 1 | 2 |
| AR | 1.0000 | 1.0000 | 1.0000 | 1.0000 | 1.0000 | 1.0000 | 1.0000 | 1.9951 | 1.1839 | 1.0000 | 1.0000 |  |
| He | 0.0000 | 0.0000 | 0.0000 | 0.0000 | 0.0000 | 0.0000 | 0.0000 | 0.4807 | 0.0381 | 0.0000 | 0.0000 |  |
| Ho | 0.0000 | 0.0000 | 0.0000 | 0.0000 | 0.0000 | 0.0000 | 0.0000 | 0.5429 | 0.0385 | 0.0000 | 0.0000 |  |
| HWE | - | - | - | - | - | - | - | NS | NS | - | - |  |
| **Lspn 019c** |  |  |  |  |  |  |  |  |  |  |  |  |
| Allelic range (bp) | 136-144 | 136-146 | 136-144 | 142-146 | 142-144 | 136 | 136 | 136-146 | 142-144 | 142-144 | 142 | 136-146 |
| Na | 3 | 4 | 3 | 2 | 2 | 1 | 1 | 3 | 2 | 2 | 1 | 4 |
| AR | 1.8590 | 2.5249 | 2.0643 | 1.1429 | 1.9610 | 1.0000 | 1.0000 | 2.8309 | 1.5256 | 1.9752 | 1.0000 |  |
| He | 0.1936 | 0.3915 | 0.2520 | 0.0286 | 0.3883 | 0.0000 | 0.0000 | 0.6166 | 0.1291 | 0.4193 | 0.0000 |  |
| Ho | 0.1379 | 0.4000 | 0.2778 | 0.0286 | 0.5152 | 0.0000 | 0.0000 | 0.7143 | 0.1373 | 0.5870 | 0.0000 |  |
| HWE | NS | NS | NS | NS | NS | - | - | NS | NS | ** | - |  |
| **Lspn 044** |  |  |  |  |  |  |  |  |  |  |  |  |
| Allelic range (bp) | 212-216 | 212-216 | 212-216 | 212-216 | 212 | 214 | 214 | 196-214 | 212-216 | 212-216 | 214-216 | 196-216 |
| Na | 3 | 3 | 3 | 3 | 1 | 1 | 1 | 3 (1) | 3 | 3 | 2 | 4 |
| AR | 2.9342 | 2.8917 | 2.8354 | 2.7933 | 1.0000 | 1.0000 | 1.0000 | 2.4190 | 2.7569 | 2.6567 | 1.9118 |  |
| He | 0.6515 | 0.6169 | 0.6021 | 0.5946 | 0.0000 | 0.0000 | 0.0000 | 0.4542 | 0.5551 | 0.4852 | 0.3302 |  |
| Ho | 0.6552 | 0.5333 | 0.5000 | 0.7143 | 0.0000 | 0.0000 | 0.0000 | 0.4286 | 0.6346 | 0.4783 | 0.4118 |  |
| HWE | NS | NS | NS | NS | - | - | - | NS | NS | NS | NS |  |
| **Lspn 094** |  |  |  |  |  |  |  |  |  |  |  |  |
| Allelic range (bp) | 202-206 | 202-206 | 202-206 | 202-204 | 202 | 200-202 | 200-202 | 202-208 | 198-204 | 180-206 | 200-202 | 180-208 |
| Na | 3 | 3 | 3 | 2 | 1 | 2 | 2 | 4 (1) | 3 | 5 (1) | 2 | 7 |
| AR | 2.5715 | 1.4746 | 2.3603 | 1.9508 | 1.0000 | 1.7953 | 1.9045 | 3.5982 | 1.7968 | 2.8371 | 1.9970 |  |
| He | 0.4428 | 0.0977 | 0.4091 | 0.3731 | 0.0000 | 0.2392 | 0.3173 | 0.7058 | 0.1944 | 0.4687 | 0.4925 |  |
| Ho | 0.4138 | 0.1000 | 0.4054 | 0.3714 | 0.0000 | 0.2727 | 0.3226 | 0.6786 | 0.2115 | 0.5000 | 0.4510 |  |
| HWE | NS | NS | NS | NS | - | NS | NS | NS | NS | NS | NS |  |
| **Pma*μ* 5** |  |  |  |  |  |  |  |  |  |  |  |  |
| Allelic range (bp) | 243-249 | 243-251 | 245-249 | 245-249 | 245-247 | 247 | 247 | 241-249 | 243-249 | 245-249 | 245 | 241-251 |
| Na | 4 | 5 (1) | 3 | 2 | 2 | 1 | 1 | 4 (1) | 4 | 2 | 1 | 6 |
| AR | 2.4618 | 2.5726 | 2.7716 | 1.9989 | 1.8866 | 1.0000 | 1.0000 | 2.2191 | 3.1922 | 1.2066 | 1.0000 |  |
| He | 0.4785 | 0.4452 | 0.6049 | 0.5056 | 0.3021 | 0.0000 | 0.0000 | 0.2682 | 0.6338 | 0.0430 | 0.0000 |  |
| Ho | 0.4828 | 0.5667 | 0.5526 | 0.4857 | 0.3636 | 0.0000 | 0.0000 | 0.1765 | 0.3333 | 0.0435 | 0.0000 |  |
| HWE | NS | NS | NS | NS | NS | - | - | * | *** | NS | - |  |
| **All loci** |  |  |  |  |  |  |  |  |  |  |  |  |
| MNA | 3.5 | 3.4 (2) | 3.8 | 2.1 | 1.5 | 1.1 | 1.1 | 2.7 (3) | 3.5 (3) | 3.1 (1) | 1.9 (3) |  |
| P | 9 | 8 | 9 | 8 | 4 | 1 | 1 | 9 | 10 | 9 | 6 |  |
| AR | 2.49 | 2.30 | 2.62 | 1.90 | 1.33 | 1.08 | 1.09 | 2.30 | 2.47 | 2.21 | 1.46 |  |
| He | 0.4069 | 0.3497 | 0.4300 | 0.3388 | 0.1101 | 0.0239 | 0.0317 | 0.4417 | 0.4275 | 0.3381 | 0.1698 |  |
| Ho | 0.4073 | 0.2846 | 0.3870 | 0.3486 | 0.1242 | 0.0273 | 0.0323 | 0.4428 | 0.3604 | 0.3423 | 0.1770 |  |

**References**

1. Van Oosterhout C, Hutchinson WF, Wills DPM, Shipley P (2004) Micro-Checker: software for identifying and correcting genotyping errors in microsatellite data. Molecular Ecology Notes 4: 535–538. doi:10.1111/j.1471-8286.2004.00684.x.
