# Supplementary material for: European Lampreys: New Insights on Postglacial Colonization, Gene Flow and Speciation
Source: PLoS One. 2016 Feb 12;11(2):e0148107. doi: 10.1371/journal.pone.0148107 (PMC4752455; doi:10.1371/journal.pone.0148107)
Supplement: S3 Table — (PDF) [file pone.0148107.s004.pdf]

Sample	Locus 1		Locus 2		Locus 3		Locus 4		Locus 5		Locus 6		Locus 7		Locus 8		Locus 9		Locus 10	
**1**	202	202	208	208	245	245	185	185	126	126	252	252	395	425	179	179	216	216	142	142
**2**	202	206	208	208	245	245	182	185	126	126	252	252	373	423	179	181	216	216	142	142
**3**	202	206	208	208	245	245	179	182	126	126	252	252	395	425	179	181	216	216	142	144
**4**	202	202	208	208	245	245	137	185	126	126	252	252	395	395	179	179	216	216	142	142
**5**	202	202	208	208	245	245	179	188	123	126	252	252	371	395	179	179	214	216	142	142
**6**	202	202	208	208	245	245	179	185	126	126	252	252	395	425	179	179	212	214	142	144
**7**	202	206	208	208	245	245	137	185	126	126	252	255	425	425	179	181	212	216	142	142
**8**	202	202	208	208	245	245	179	185	126	126	252	252	395	395	179	179	216	216	142	144
**9**	202	202	208	208	245	245	179	185	126	126			423	423	179	179	212	216	142	144
**10**	202	206	208	208	245	249	179	179	123	126	252	282	395	425	179	181	216	216	142	144
**11**	180	202	208	208	245	245	137	185	126	126	252	252	395	395	179	179	216	216	142	144
**12**	202	202	208	208	245	245	179	188	123	126	252	258	395	395	179	179	214	216	142	142
**13**	202	202	208	208	245	245	182	185	126	126	252	255	371	395	179	179	216	216	142	144
**14**	202	202	208	208	245	245	182	185	126	126	252	252	395	425	179	181	214	214	142	144
**15**	202	202	208	208	245	245	179	185	126	126	252	252	395	425	179	181	216	216	142	144
**16**	202	204	208	208	245	245	179	185	123	126	252	258	395	425	179	179	216	216	142	144
**17**	202	202	208	208	245	245	185	185	126	126	252	255	395	395	181	181	212	216	142	144
**18**	202	202	208	208	245	245	185	185	126	126	252	252	373	425	179	179	214	216	142	142
**19**	202	202	208	208	245	245	137	185	126	126	252	258	373	425	179	179	214	216	142	144
**20**	202	204	208	208	245	245	185	185	126	126	252	252	395	425	179	179	214	216	142	144
**21**	204	204	208	208	245	245	179	185	126	126	252	258	423	423	179	181	216	216	142	144
**22**	202	204	208	208	245	245	179	179	126	126	252	252	395	395	179	179	212	216	142	144
**23**	202	204	208	208	245	249	179	185	126	126	252	252	395	395	179	179	212	216	142	142
**24**	202	204	208	208	245	245	179	185	126	126	252	252	395	425	179	179	212	216	142	142
**25**	202	206	208	208	245	245	179	185	126	126	252	252	373	395	179	181	214	216	142	144
**26**	202	202	208	208	245	245	179	185	126	126	252	252	395	425	179	179	212	216	142	144
**27**	202	202	208	208	245	245	185	185	126	126	282	282	395	395	179	179	214	216	142	142
**28**	198	202	208	208	245	245	179	179	126	126	255	255	395	423	179	181	216	216	142	142
**29**	202	204	208	208	245	245	179	179	126	126			425	425	179	179	216	216	142	142
**30**	204	206	208	208	245	245	179	182	123	126	252	252	395	425	179	181	212	212	142	144
**31**	180	206	208	208	245	245	185	185	126	126	252	252	395	395	179	179	212	216	142	142
**32**	202	202	208	208	245	245	179	185	126	126	252	258	395	423	179	179	212	212	142	144
**33**	202	204	208	208	245	245	179	179	126	126	252	252	395	423	179	179	216	216	142	142
**34**	202	202	208	208	245	245	179	179	126	126	252	252	395	395	179	179	212	216	142	144
**35**	202	204	208	208	245	245	179	179	126	126	252	255	395	395	179	179	212	216	142	144
**36**	202	204	208	208	245	245	137	179	126	126	252	252	395	423	179	179	212	216	142	144
**37**	202	202	208	208	245	245	179	179	126	126	252	252	395	423	179	179	216	216	142	142
**38**	202	204	208	208	245	245	179	188	126	126	252	252	423	423	179	179	216	216	142	144
**39**	202	204	208	208	245	245	179	179	123	126	252	252	395	395	179	179	216	216	142	144
**40**	202	202	208	208	245	245	185	185	126	126	252	252	395	423	179	179	216	216	142	142
**41**	180	202	208	208	245	245	137	185	126	126	252	252	395	425	179	179	216	216	142	144
**42**	202	204	208	208	245	245	185	185	126	126	252	252	395	395	179	181	216	216	142	144
**43**	202	202	208	208	245	245	179	185	123	126	252	252	395	425	179	179	214	216	142	144
**44**	202	204	208	208	245	245	185	185	126	126	252	252	373	425	179	179	212	216	142	142
**45**	202	202	208	208	245	245	185	185	126	126	252	252	373	395	179	179	214	216	142	142
**46**	202	202	208	208	245	245	179	182	126	126	252	252	395	395	179	181	216	216	142	142
**47**	202	204	208	208	245	247	188	188	123	126	255	255	395	423	179	181	216	216	142	142
**48**	202	202	208	208	247	247	185	188	123	123	255	255	395	395	179	181	214	216	142	142
**49**	202	202	208	208	243	243	182	182	123	123	255	255	395	395	179	181	216	216	142	142
**50**	202	202	208	208			185	188			255	255	391	395	179	181	212	216		
**51**	198	202	208	208	247	247	188	188	123	123	252	255	389	395	179	181	214	216	142	142
**52**	202	202	208	208	247	247	185	188	126	126	255	255	389	425	179	181	214	216	142	142
**53**	202	202	208	208	249	249	185	191	120	120	255	255	369	389	179	179	214	216	142	142
**54**	202	202	208	208	245	245	173	185	123	126	255	255	395	395	179	181	212	216	142	142
**55**	198	202	208	208					123	123	255	255	373	395	181	181	212	216	142	142
**56**	202	202	208	208			185	185	123	123	255	255	389	391	179	181	216	216	142	142
**57**	202	202	208	208	247	247	188	188	123	123	255	255	395	395	179	181	216	216	142	142
**58**	202	202	208	208	247	247	185	188	123	123	255	255	391	395	179	181	214	216	142	142
**59**	202	202	208	208	247	247	188	188	123	123	255	255	395	395	179	179	216	216	142	142
**60**	202	204	208	208	245	247	182	182	123	126	255	255	389	389	181	181	216	216	142	142
**61**	202	202	208	208	245	245	185	188	120	123	252	255	389	389	179	181	214	216	142	142
**62**	202	202	208	208	249	249	191	191			255	255	389	425	179	181	214	216	142	142
**63**	202	202	208	208	245	249	188	188	120	126	252	255	389	391	179	181	214	216	142	142
**64**	202	202	204	208	249	249	191	191	123	123	255	255	389	389	179	181	214	216	142	142
**65**	202	202	208	208	247	247	191	191	120	123	252	255	389	389	179	181	212	216	142	142
**66**	202	202	208	208	247	247	191	191	120	123	252	255	389	389	179	181	214	216	142	144
**67**	202	202	208	208	247	249	182	182	120	126	255	255	389	389	179	181	216	216	142	142
**68**	202	202	208	208	245	245	185	185	120	120	252	255	389	389	179	181	216	216	142	144
**69**	202	202	208	208	247	249	191	191	126	126	255	255	389	389	179	181	214	216	142	142
**70**	202	204	208	208	245	247	185	191	123	126	255	255	389	389	179	181	214	216	142	142
**71**	202	204	208	208	245	245	185	185	123	126	255	255	389	389	179	181	212	214	142	142
**72**	202	204	208	208	247	247	188	188	120	126	255	255	389	389	179	181	214	216	142	142
**73**	202	204	204	208	245	247	185	188	120	123	255	255	389	395	179	181	216	216	142	142
**74**	202	202	208	208	249	249	191	191	120	126	255	255	389	389	179	181	212	214	142	142
**75**	202	202	208	208	245	245	185	191	126	126	252	255	389	389	179	181	212	214	142	142
**76**	202	202	208	208	245	245	188	188	120	123	255	255	389	389	179	181	214	216	142	142
**77**	202	202	208	208	247	247	185	185	126	126	255	255	389	395	179	179	214	216	142	142
**78**	202	202	208	208	245	247	185	191	120	123	252	255	389	389	179	181	214	216	142	142
**79**	202	202	208	208	245	245	188	188	126	126	255	255	389	389	179	181	216	216	142	142
**80**	202	202	208	208	247	247	191	191	123	123	252	255	389	389	179	181	212	216	142	142
**81**	202	202	208	208	245	247	182	191	123	126	255	255	389	391	179	179	214	216	142	142
**82**	202	202	208	208	245	247	182	182	120	123	252	255	389	395	179	181	216	216	142	142
**83**	202	202	208	208	245	247	188	188	120	126	252	255	389	389	179	181	214	216	142	144
**84**	202	204	208	208	245	247	191	191	123	126	255	255	389	389	179	179	214	214	142	142
**85**	202	202	208	208	249	249	185	191	120	120	255	255	389	389	179	181	216	216	142	142
**86**	202	202	208	208	245	245	179	185	120	123	255	255	391	395	179	179	212	216	142	142
**87**	202	202	208	208	243	247	185	185	123	123	255	255	391	395	179	181	212	216	142	142
**88**	202	202	208	208	243	247	188	188	123	123	255	255	391	391	179	179	216	216	142	142
**89**	202	202	208	208	247	247	179	182	123	123	255	255	389	391	179	181	216	216	142	144
**90**	202	202	208	208	247	247	185	185	123	123	255	255	395	395	179	181	216	216	142	144
**91**	202	204	208	208	247	247	188	188	123	126	255	255	389	391	179	181	214	216	142	142
**92**	202	204	208	208	243	247	188	188	123	123	255	255			179	179	212	216	142	144
**93**	202	202	208	208	247	247	179	179	123	126	252	255	395	395	179	179	212	212	142	142
**94**	202	202	208	208			185	185	123	123	255	255	389	395	179	181	216	216	142	144
**95**	202	202	208	208	245	247	188	188	123	123	255	258	395	395	179	181	214	216	142	142
**96**	202	202	208	208	247	247	188	188	123	123			389	391	179	181	212	214	142	142
**97**	202	202	208	208	247	247	179	182	123	123	252	255	391	391	179	181	212	216	142	142
**98**	202	202	208	208	245	245	179	179	123	126	255	255	395	425	179	181	216	216	142	142
**99**	200	200	208	208	245	245	125	125	126	126	249	252	373	373	179	179	214	216	142	142
**100**	202	202	208	208	245	245	125	125	126	126	249	252	373	373	179	179	216	216	142	142
**101**	200	202	208	208	245	245	125	125	126	126	249	252	373	373	179	179	214	216		
**102**	200	200	208	208	245	245	125	125	126	126	249	252	373	373	179	179	216	216		
**103**	202	202	208	208	245	245	125	125	126	126	252	252	373	373	179	179	216	216		
**104**	200	200	208	208	245	245	125	125	126	126	249	252	373	373	179	179	214	216		
**105**	200	202	208	208	245	245	125	125	126	126	249	252	373	373	179	179	214	216	142	142
**106**	200	202	208	208	245	245	182	185	126	126	249	252	373	451	179	179	214	216		
**107**	200	202	208	208	245	245			126	126	249	249	373	451	179	179	216	216	142	142
**108**	200	202	208	208	245	245	179	185	126	126	249	252	373	373	179	179	216	216	142	142
**109**	200	200	208	208	245	245	125	125	123	126	249	252	373	373	179	179	216	216	142	142
**110**	200	200	208	208	245	245	125	125	126	126	249	249	373	373	179	179	216	216		
**111**	200	200	208	208	245	245	125	185	126	126	249	252	373	373	179	179	216	216	142	142
**112**	200	200	208	208	245	245			126	126	249	249	373	373	179	179	216	216	142	142
**113**	202	202	208	208	245	245	125	188	126	126	249	252	373	373	179	179	216	216	142	142
**114**	200	202	208	208	245	245	125	125	126	126	249	252	373	373	179	179	216	216	142	142

**115**	200	200	208	208	245	245	125	125	126	126	249	252	373	373	179	179	216	216	142	142
**116**	200	202	208	208	245	245	125	125	126	126	249	252	373	373	179	179	216	216	142	142
**117**	200	200	208	208	245	245	125	185	126	126	249	252	373	373	179	179	216	216	142	142
**118**	202	202	208	208	245	245	125	125	126	126	249	252	373	373	179	179	214	216	142	142
**119**	200	202	208	208	245	245			126	126	249	252	373	373	179	179	216	216		
**120**	200	202	208	208	245	245	125	125	126	126	252	252	373	373	179	179	214	216		
**121**	200	200	208	208	245	245	125	125	126	126	249	252	373	451	179	179	216	216		
**122**	202	202	208	208	245	245	125	125	126	126	249	252	373	373	179	179	216	216	142	142
**123**	200	200	208	208	245	245			126	126	249	252	373	373	179	179	216	216		
**124**	200	202	208	208	245	245			126	126	252	252	373	373	179	179	216	216	142	142
**125**	200	202	208	208	245	245			126	126	249	249	373	373	179	179	214	216	142	142
**126**	202	202	208	208	245	245	125	125	126	126	249	249	373	373	179	179	216	216	142	142
**127**	200	202	208	208	245	245	125	125	126	126	249	249	373	373	179	179	216	216	142	142
**128**	200	200	208	208	245	245	125	125	126	126	252	252	373	373	179	179	216	216	142	142
**129**	200	200	208	208	245	245	125	125	126	126	249	252	373	373	179	179	216	216	142	142
**130**	202	202	208	208	245	245	125	125	126	126	249	252	373	451	179	179	214	216	142	142
**131**	200	200	208	208	245	245			126	126	249	252	373	373	179	179	214	216		
**132**	202	202	208	208	245	245	125	125	126	126	249	249	373	373	179	179	214	216	142	142
**133**	200	202	208	208	245	245	125	125	126	126	249	252	373	373	179	179	214	216		
**134**	200	202	208	208	245	245	125	125	126	126	249	249	373	373	179	179	214	216		
**135**	200	202	208	208	245	245			126	126	252	252	373	373	179	179	214	216	142	142
**136**	200	202	208	208	245	245	125	125	126	126	249	252	373	373	179	179	214	216		
**137**	200	202	208	208	245	245	125	125	126	126	249	252	373	373	179	179	216	216	142	142
**138**	200	202	208	208	245	245	125	125	126	126	249	249	373	373	179	179	216	216	142	142
**139**	200	202	208	208	245	245			126	126	249	252	373	373	179	179	214	216	142	142
**140**	200	200	208	208	245	245			126	126	249	252	373	373	179	179	214	216		
**141**	200	202	208	208	245	245			126	126	249	249	373	451	179	179	214	216	142	142
**142**	200	202	208	208	245	245	125	125	126	126	249	249	373	373	179	179	216	216		
**143**	200	200	208	208	245	245	125	125	123	123	249	252	373	373	179	179	216	216	142	142
**144**	202	202	208	208	245	245			126	126	249	252	373	373	179	179	214	216	142	142
**145**	200	202	208	208	245	245			126	126	249	252	373	373	179	179	216	216	142	142
**146**	200	202	208	208	245	245			123	126	252	252	373	373	179	179	216	216	142	142
**147**	200	200	208	208	245	245	125	125	123	126	249	249	373	373	179	179	216	216	142	142
**148**	200	200	208	208	245	245			126	126	249	249	373	373	179	179	214	216	142	142
**149**	202	202	208	208	245	245			126	126	252	252	373	373	179	179	214	216	142	142
**150**	204	206	204	208			182	182	120	123	252	252	391	395	179	181	212	212	136	146
**151**	202	208	204	204	247	247	185	185	120	123	252	252	375	395	179	181	212	214	136	146
**152**	202	204	204	208	247	247	182	185	120	123	252	252	391	391	179	179	196	212	142	146
**153**	202	208	204	208	247	247	182	182	120	123	252	252	391	395	179	179	196	212	136	146
**154**			204	204	247	247	185	185	123	123	252	252	391	395	179	179	212	212	142	146
**155**	206	208	204	208	247	247	182	182	120	123	252	252	395	395	179	181	196	196	136	146
**156**	202	204	204	208	247	247	185	185	120	123	252	252	375	391	179	181	212	212	142	146
**157**	202	208	208	208	247	247	182	185	120	123	252	252	391	415	179	179	212	212	146	146
**158**			204	208	245	247	185	185	120	120	252	252	391	395	179	181	212	212	142	146
**159**	202	204	204	204	247	247	182	185	120	123	252	252	375	395	179	179	212	212	136	146
**160**	206	208	204	204	247	247	182	185	120	123	252	252	375	391	179	179	212	212	136	146
**161**	202	202	204	208	247	247	182	185	120	120	252	252	391	391	179	179	196	212	136	136
**162**			204	204	245	247	185	185	123	123	252	252			179	181	196	212	136	146
**163**	202	208	204	208	247	247	185	185	120	120	252	252	395	415	179	179	212	212	136	136
**164**	202	206	204	204	247	247	182	185	120	120	252	252	391	395	179	181	212	212	142	146
**165**	204	208	204	208	247	249			120	123	252	252	391	395	179	181	212	212	146	146
**166**	202	202	204	208	247	247	182	185	123	123	252	252	375	391	179	179	212	212	146	146
**167**	202	204	204	208	247	247	182	182	120	123	252	252	391	391	179	179	196	196	136	146
**168**	206	206	208	208	247	247	182	182	120	120	252	252	375	391	179	179	196	212	146	146
**169**	202	208	204	208	247	247	185	185	120	123	252	252	391	395	179	181	212	212	136	146
**170**			204	208	241	247	185	185	120	123	252	252	391	395	179	179	212	214	136	146
**171**			208	208	247	247	182	185	120	120	252	252	391	395	179	179	212	212	136	142
**172**	202	206	204	208	241	247	182	185	123	123			375	391	179	179	196	212	136	146
**173**	202	202	204	204	247	247	182	182	120	123	252	252	395	395	179	179	196	212	136	146
**174**	204	208	208	208	249	249	185	185	123	123	252	252	391	395	179	179	212	212	142	146
**175**	202	202	204	204	247	247	182	185	123	123	252	252	391	391	179	179	196	212	136	146
**176**	202	208	204	204	247	247	182	182	120	120	252	252	391	395	179	179	196	212	146	146
**177**	202	202	204	204	241	247	185	185	120	120			395	395	179	179	196	212	136	136
**178**	204	208	204	208	247	247	185	185	120	123	252	252	391	391	179	179	196	212	136	146
**179**			204	208	247	247	182	185	120	123	252	252	391	391	179	179	214	214	142	142
**180**	208	208	204	204	247	247	182	185	120	120	252	252	391	391	179	181	196	212	142	146
**181**	202	202	204	208	247	247	185	185	120	123	252	252	395	395	179	181	212	212	146	146
**182**			204	208	247	247	182	182	120	123	252	252	391	395	179	179	212	212	136	142
**183**	204	208	204	204	247	247	182	185	120	123	252	252	391	415	179	179	196	212	136	146
**184**	208	208	204	208	249	249	182	182	120	120	252	252	391	395	179	179	212	212	136	146
**185**	202	202	208	208	245	247	185	185	123	123	255	255	395	395	179	179	212	212	142	142
**186**	202	202	208	208	245	247	185	185	123	123	255	255	395	395	179	179	212	212	142	142
**187**	202	202	208	208	245	245	185	185	123	123	255	255	395	395	179	179	212	212	142	142
**188**	202	202	208	208	245	245	185	185	123	123	255	255	395	395	179	179	212	212	142	144
**189**	202	202	208	208	245	247	185	185	123	123	255	255	395	395	179	179	212	212	142	144
**190**	202	202	208	208	245	245	185	185	123	123	255	255	395	425	179	179	212	212	142	142
**191**	202	202	208	208	245	245	185	185	123	123	255	255	395	425	179	179	212	212	142	142
**192**	202	202	208	208	245	247	185	185	123	123	255	255	395	395	179	179	212	212	142	144
**193**	202	202	208	208	245	245	185	185	123	123	255	255	395	395	179	179	212	212	142	144
**194**	202	202	208	208	245	247	185	185	123	123	255	255	395	425	179	179	212	212	142	144
**195**	202	202	208	208	245	245	185	185	123	123	255	255	395	423	179	179	212	212	142	142
**196**	202	202	208	208	245	245	185	185	123	123	255	255	395	395	179	179	212	212	142	144
**197**	202	202	208	208	245	247	185	185	123	123	255	255	395	423	179	179	212	212	142	142
**198**	202	202	208	208	245	245	185	185	123	123	255	282	395	395	179	179	212	212	142	144
**199**	202	202	208	208	245	247	185	185	123	123	255	255	395	395	179	179	212	212	142	142
**200**	202	202	208	208	245	245	185	185	123	123	255	255	395	395	179	179	212	212	142	142
**201**	202	202	208	208	245	247	185	185	123	123	255	255	425	425	179	179	212	212	142	144
**202**	202	202	208	208	245	245	185	185	123	123	255	255	395	395	179	179	212	212	142	144
**203**	202	202	208	208	245	245	185	185	123	123	255	255	395	395	179	179	212	212	142	144
**204**	202	202	208	208	245	245	185	185	123	123	255	255	395	395	179	179	212	212	142	142
**205**	202	202	208	208	245	247	185	185	123	123	255	255	425	425	179	179	212	212	142	144
**206**	202	202	208	208	245	247	185	185	123	123	255	282	395	395	179	179	212	212	142	144
**207**	202	202	208	208	245	245	185	185	123	123	255	255	395	395	179	179	212	212	142	144
**208**	202	202	208	208	245	245	185	185	123	123	255	255	395	395	179	179	212	212	142	142
**209**	202	202	208	208	245	245	185	185	123	123	255	255	395	425	179	179	212	212	142	142
**210**	202	202	208	208	245	245	185	185	123	123	255	255	395	395	179	179	212	212	142	144
**211**	202	202	208	208	245	247	185	185	123	123	255	255	395	395	179	179	212	212	142	144
**212**	202	202	208	208	245	245	185	185	123	123	255	255	395	425	179	179	212	212	142	144
**213**	202	202	208	208	245	245	185	185	123	123	255	255	395	425	179	179	212	212	142	142
**214**	202	202	208	208	245	247	185	185	123	123	255	255	395	425	179	179	212	212	142	142
**215**	202	202	208	208	245	245	185	185	123	123	255	255	395	395	179	179	212	212	142	144
**216**	202	202	208	208	245	245	185	185	123	123	255	255	395	395	179	179	212	212	142	142
**217**	202	202	208	208	245	245	185	185	123	123	255	255	395	425	179	179	212	212	142	142
**218**	202	202	208	208	247	247	188	188	123	123	252	252	379	379	179	179	214	214	136	136
**219**	200	202	208	208	247	247	188	188	123	123	252	252	379	379	179	179	214	214	136	136
**220**	202	202	208	208	247	247	188	188			252	252	379	379	179	179	214	214	136	136
**221**	202	202	208	208	247	247	188	188			252	252	379	379	179	179	214	214	136	136
**222**	202	202	208	208	247	247	188	188	123	123	252	252	379	379	179	179	214	214	136	136
**223**	202	202	208	208	247	247	188	188			252	252	379	379	179	179	214	214	136	136
**224**	200	202	208	208	247	247	188	188	123	123	252	252	379	379	179	179	214	214	136	136
**225**	202	202	208	208	247	247	188	188	123	123	252	252	379	379	179	179	214	214	136	136
**226**	202	202	208	208	247	247	188	188			252	252	379	379	179	179	214	214	136	136
**227**	202	202	208	208	247	247	188	188	123	123	252	252	379	379	179	179	214	214	136	136
**228**	200	202	208	208	247	247	188	188	123	123	252	252	379	379	179	179	214	214	136	136
**229**	202	202	208	208	247	247	188	188	123	123	252	252	379	379	179	179	214	214	136	136

**230**	202	202	208	208	247	247	188	188			252	252	379	379	179	179	214	214	136	136
**231**	202	202	208	208	247	247	188	188			252	252	379	379	179	179	214	214	136	136
**232**	202	202	208	208	247	247	188	188	123	123	252	252	379	379	179	179	214	214	136	136
**233**	202	202	208	208	247	247	188	188			252	252	379	379	179	179	214	214	136	136
**234**	202	202	208	208	247	247	188	188			252	252	379	379	179	179	214	214	136	136
**235**	202	202	208	208	247	247	188	188	123	123	252	252	379	379	179	179	214	214	136	136
**236**	202	202	208	208	247	247	188	188	123	123	252	252	379	379	179	179	214	214	136	136
**237**	200	202	208	208	247	247	188	188	123	123	252	252	379	379	179	179	214	214	136	136
**238**	202	202	208	208	247	247	188	188			252	252	379	379	179	179	214	214	136	136
**239**	200	202	208	208	247	247	188	188	123	123	252	252	379	379	179	179	214	214	136	136
**240**	202	202	208	208	247	247	188	188	123	123	252	252	379	379	179	179	214	214	136	136
**241**	202	202	208	208	247	247	188	188	123	123	252	252	379	379	179	179	214	214	136	136
**242**	200	202	208	208	247	247	188	188	123	123	252	252	379	379	179	179	214	214	136	136
**243**	202	202	208	208	247	247	188	188			252	252	379	379	179	179	214	214	136	136
**244**	202	202	208	208	247	247	188	188	123	123	252	252	379	379	179	179	214	214	136	136
**245**	202	202	208	208	247	247	188	188			252	252	379	379	179	179	214	214	136	136
**246**	200	202	208	208	247	247	188	188			252	252	379	379	179	179	214	214	136	136
**247**	200	202	208	208	247	247	188	188			252	252	379	379	179	179	214	214	136	136
**248**	202	202	208	208	247	247	188	188			252	252	379	379	179	179	214	214	136	136
**249**	202	202	208	208	247	247	188	188			252	252	379	379	179	179	214	214	136	136
**250**	200	202	208	208	247	247	188	188			252	252	379	379	179	179	214	214	136	136
**251**	200	200	208	208	247	247	188	188	123	123	252	252	379	379	179	179	214	214	136	136
**252**	200	200	208	208	247	247	188	188			252	252	379	379	179	179	214	214	136	136
**253**	202	202	208	208	247	247	188	188			252	252	379	379	179	179	214	214	136	136
**254**	200	200	208	208	247	247	188	188			252	252	379	379	179	179	214	214	136	136
**255**	200	202	208	208	247	247	188	188			252	252	379	379	179	179	214	214	136	136
**256**	200	202	208	208	247	247	188	188			252	252	379	379	179	179	214	214	136	136
**257**	200	202	208	208	247	247	188	188			252	252	379	379	179	179	214	214	136	136
**258**	200	200	208	208	247	247	188	188			252	252	379	379	179	179	214	214	136	136
**259**	200	200	208	208	247	247	188	188	123	123	252	252	379	379	179	179	214	214	136	136
**260**	200	200	208	208	247	247	188	188	123	123	252	252	379	379	179	179	214	214	136	136
**261**	200	202	208	208	247	247	188	188	123	123	252	252	379	379	179	179	214	214	136	136
**262**	200	200	208	208	247	247	188	188			252	252	379	379	179	179	214	214	136	136
**263**	200	202	208	208	247	247	188	188			252	252	379	379	179	179	214	214	136	136
**264**	200	202	208	208	247	247	188	188			252	252	379	379	179	179	214	214	136	136
**265**	200	200	208	208	247	247	188	188			252	252	379	379	179	179	214	214	136	136
**266**	200	200	208	208	247	247	188	188	123	123	252	252	379	379	179	179	214	214	136	136
**267**	200	200	208	208	247	247	188	188			252	252	379	379	179	179	214	214	136	136
**268**	200	200	208	208	247	247	188	188	123	123	252	252	379	379	179	179	214	214	136	136
**269**	200	200	208	208	247	247	188	188			252	252	379	379	179	179	214	214	136	136
**270**	200	202	208	208	247	247	188	188			252	252	379	379	179	179	214	214	136	136
**271**	200	200	208	208	247	247	188	188	123	123	252	252	379	379	179	179	214	214	136	136
**272**	200	200	208	208	247	247	188	188			252	252	379	379	179	179	214	214	136	136
**273**	200	200	208	208	247	247	188	188			252	252	379	379	179	179	214	214	136	136
**274**	200	200	208	208	247	247	188	188			252	252	379	379	179	179	214	214	136	136
**275**	200	200	208	208	247	247	188	188			252	252	379	379	179	179	214	214	136	136
**276**	200	202	208	208	247	247	188	188	123	123	252	252	379	379	179	179	214	214	136	136
**277**	200	200	208	208	247	247	188	188			252	252	379	379	179	179	214	214	136	136
**278**	200	202	208	208	247	247	188	188	123	123	252	252	379	379	179	179	214	214	136	136
**279**	200	200	208	208	247	247	188	188			252	252	379	379	179	179	214	214	136	136
**280**	200	202	208	208	247	247	188	188			252	252	379	379	179	179	214	214	136	136
**281**	200	200	208	208	247	247	188	188	123	123	252	252	379	379	179	179	214	214	136	136
**282**	204	204	208	208	249	249	137	185	126	126	252	258	395	425	179	179	212	216	142	142
**283**	202	206	208	208	249	249	179	185	126	126	246	252	375	395	179	181	212	216	142	142
**284**	202	204	208	208	245	249	182	188	126	126	252	252	395	395	179	179	212	214	136	136
**285**	202	202	208	208	249	249	179	185	123	126	252	252	371	395	179	179	214	216	142	142
**286**	202	202	208	208	245	249	185	185	126	126	246	255	375	395	179	179	214	214	142	142
**287**	202	202	208	208	249	249	179	185	123	126	252	258	395	425	179	179	212	216	142	142
**288**	202	202	208	208	245	249	179	188	126	126	246	252	375	395	179	179	212	216	142	142
**289**	202	202	208	208	245	249	179	179	126	126	252	252	395	425	179	179	212	216	142	142
**290**	202	204	208	208	245	249	179	185	126	126	246	252	395	395	179	179	212	216	136	142
**291**	202	202	208	208	245	249	137	185	126	126	246	252	375	395	179	179	214	214	142	142
**292**	202	202	208	208	249	249	182	185	126	126	252	258	395	395	179	179	214	214	142	142
**293**	202	206	208	208	249	249	185	185	126	126	252	252	395	415	179	179	216	216	142	142
**294**	202	202	208	208	249	249	179	188	123	126	246	246	395	425	179	179	212	214	142	144
**295**	202	204	208	208	247	249	185	185	126	126	252	267	393	395	179	179	212	214	142	142
**296**	202	202	208	208	249	249	185	185	123	126	246	246	395	415	179	179	216	216	142	142
**297**	202	204	208	208	245	249	179	179	126	126	246	252	395	415	179	179	216	216	136	142
**298**	202	202	208	208	245	249	179	185	123	126	252	258	395	395	179	181	212	216	142	142
**299**	202	202	208	208	249	249	185	188	123	126	252	258	395	415	179	181	212	212	142	144
**300**	204	204	208	208	249	249	179	182	126	129	246	246	375	425	179	179	214	216	142	142
**301**	202	206	208	208	243	249	179	188	126	126	252	252	395	395	179	179	212	214	142	142
**302**	202	204	208	208	245	247	179	179	123	126			375	395	179	179	212	216	142	142
**303**	202	202	208	208	249	249	179	188	126	126	252	258	375	395	179	179	214	216	142	142
**304**	202	206	208	208	245	249	137	185	126	126	252	252	395	415	179	179	216	216	142	142
**305**	202	202	208	208	245	249	185	188	126	126	246	252	395	425	179	179	214	216	142	142
**306**	202	202	208	208	249	249	179	179	126	126			395	395	179	179	212	216	142	142
**307**	202	206	208	208	245	245	185	185	123	126	252	252	371	375	179	179	212	216	142	142
**308**	202	204	208	208	245	245	185	185	126	126	252	252	375	395	179	179	216	216	142	142
**309**	202	206	208	208	249	249	179	185	126	126	246	252	375	393	179	181	216	216	142	142
**310**	202	202	208	208	245	249	185	185	126	126	252	252	395	395	179	179	214	216	142	142
**311**	202	202	208	208	249	249	191	191	126	126	252	258	395	395	179	181	212	214	142	142
**312**	202	204	208	208	249	249	137	191	123	123	246	246	395	395	179	181	214	214	142	142
**313**	202	204	208	208	245	245	137	185	123	126	252	252	395	395	181	181	212	212	142	142
**314**	204	204	208	208	245	249	191	191	123	126	252	252	395	395	181	181	214	214	142	142
**315**	202	204	208	208	249	249	137	191	123	123	252	252	395	395	181	181	212	214	142	142
**316**	202	204	208	208	249	249	137	137	123	126	246	252	395	395	179	181	212	214	142	142
**317**	204	204	208	208	249	249	191	191	123	126	252	252	395	395	179	181	214	216	142	142
**318**	204	204	208	208	245	249	191	191	123	123	246	246	395	395	179	181	212	214	142	142
**319**	204	204	208	208	245	249	191	191	123	123	252	252	395	395	179	181	212	216	142	142
**320**	202	202	208	208	245	245	185	191	123	126	252	252	395	395	181	181	212	214	142	142
**321**	204	204	208	208	245	249	185	191	123	123	246	252	395	395	179	181	214	214	142	142
**322**	204	204	208	208	245	249	191	191	123	123	246	246	395	395	181	181	214	216	142	142
**323**	202	204	208	208	245	249	137	191	123	123	246	252	395	395	179	181	212	214	142	142
**324**	202	204	208	208	245	245	185	191	123	123	252	252	395	395	181	181	212	214	142	142
**325**	204	204	208	208	245	249	191	191	123	126	252	252	395	395	179	181	212	214	142	142
**326**	202	204	208	208	249	249	137	137	123	126	246	252	395	395	179	181	212	214	142	142
**327**	202	204	208	208	245	249	191	191	123	123	246	246	395	395	181	181	214	216	142	142
**328**	202	204	208	208	249	249	137	137	123	126	252	252	395	395	179	181	212	214	142	142
**329**	202	204	208	208	245	249	137	185	123	123	252	252	395	395	181	181	214	214	142	142
**330**	204	204	208	208	245	245	137	137	123	126	252	252	395	395	179	181	214	216	142	142
**331**	202	204	208	208	245	249	137	191	123	123	252	252	395	395	179	181	214	216	142	142
**332**	204	204	208	208	245	249	137	191	123	123	252	252	395	395	181	181	212	214	142	142
**333**	202	204	208	208	249	249	137	191	123	126	246	252	395	395	179	181	212	214	142	142
**334**	204	204	208	208	245	245	137	191	123	123	252	258	395	395	179	181	214	214	142	142
**335**	204	204	208	208	245	249	185	191	123	123	252	252	395	395	179	179	212	214	142	142
**336**	204	204	208	208	245	249	137	185	123	123	252	258	395	395	181	181	216	216	142	142
**337**	204	204	208	208	245	249	137	191	123	126	252	252	395	395	179	179	212	214	142	146
**338**	204	204	208	208	249	249	137	137	123	126	246	252	395	395	179	181	212	214	142	142
**339**	204	204	208	208	249	249	137	137	123	123	252	252	395	395	179	181	214	214	142	142
**340**	204	204	208	208	245	249	137	191	123	126	252	252	395	395	179	181	214	216	142	142
**341**	204	204	208	208	245	245	191	191	123	123	246	252	395	395	179	181	214	214	142	142
**342**	204	204	208	208	245	245	137	185	123	123	246	252	395	395	179	181	212	214	142	142
**343**	204	204	208	208	245	249	137	191	123	126	246	252	395	395	179	181	212	214	142	142
**344**	204	204	208	208	245	249	137	137	123	123	252	252	395	395	181	181	212	212	142	142

**345**	202	204	208	208	245	245	185	185	123	123	252	252	395	395	179	181	214	216	142	142
**346**	202	206	208	208	245	249	137	185	126	126			371	395	179	181	216	216	142	142
**347**	202	202	208	208	249	249	179	179	126	126	252	252	395	425	179	179	214	214	142	142
**348**	202	202	208	208	245	249	179	185	126	126	246	252	395	395	179	181	214	216	142	144
**349**	202	202	208	208	245	249	179	191	126	126	246	246	375	395	179	181	212	212	142	142
**350**	202	202	208	208	245	249	185	185	126	126	252	258	395	425	179	179	214	216	142	142
**351**	202	206	208	208	247	249	185	185	123	126	246	267	395	395	179	181	212	216	142	144
**352**	202	202	208	208	245	245	137	179	123	126	252	252	375	395	179	179	216	216	142	142
**353**					245	249			123	126			395	395						
**354**									126	126			375	395						
**355**					247	247			123	126			373	425						
**356**	202	202	208	208	245	249	185	188	126	126	252	267	375	425	179	181	214	216	142	142
**357**	202	202	208	208	245	249	137	185	126	126	252	252	395	395	179	179	214	214	142	142
**358**	202	204	208	208	249	249	185	185	126	126	267	282	375	425	179	179	216	216	142	142
**359**	202	204	208	208	247	249	185	185	126	126	252	258	375	395	179	179	212	216	136	142
**360**	204	204							123	126			395	395	181	181				
**361**	202	204	208	208	245	245	137	185	126	129	246	252	393	395	179	179	214	216	142	142
**362**	202	204	208	208	245	247	185	185	126	126	252	258	375	395	179	179	216	216	142	142
**363**	202	202	208	208	245	245	185	185	126	126	267	267	393	395	179	181	214	214	142	142
**364**	202	204	208	208	245	245	185	191	123	126	252	252	375	395	179	179	216	216	142	142
**365**	202	204	208	208	249	249	182	185	123	126	252	252	395	395	179	179	216	216	142	142
**366**	204	204	208	208	245	249	179	182	126	126	252	252	375	395	179	179	216	216	142	142
**367**	202	202	208	208	245	249	137	191	126	126	252	252	395	395	179	179	216	216	142	144
**368**	202	204	208	208	245	249	137	185	126	126			371	395	179	179	214	214	142	142
**369**	202	204	208	208	247	249	137	179	123	126	252	258	425	425	179	179	214	216	142	142
**370**	202	202	208	208	245	245	185	191	126	126	252	258	395	395	179	179	214	216	142	142
**371**	202	204	208	208	245	247	137	182	126	126	252	258	395	395	179	179	212	214	142	144
**372**	202	202	208	208	245	249	137	185	126	126			395	423	179	179	212	216	142	144
**373**	202	202	208	208	249	249	185	185	126	126	252	252	393	395	179	179	216	216	142	142
**374**	202	202	208	208	249	249	179	185	126	126	252	252	395	395	179	179	214	216	142	142
**375**	202	202	208	208	245	249	137	185	126	126	252	267	395	395	179	179	214	216	142	142
**376**	202	202	208	208	249	249	182	182	123	126	246	246	375	425	179	179	216	216	142	144
**377**	202	202	208	208	249	249	137	185	126	126	267	267	375	425	179	179	212	216	142	142
**378**	202	206	208	208	249	249	182	191	123	126	252	252	395	395	179	179	212	214	136	142
**379**	202	202	208	208	249	249	185	185	126	126	297	297	395	395	179	181	212	216	136	142
**380**	202	202	208	208	247	249	137	137	126	126	252	258	395	395	179	181	212	214	142	142
**381**	202	206	208	208	245	245	137	191	126	126	267	267	393	395	179	179	216	216	142	142
**382**	202	204	208	208	247	249	185	191	126	126	252	252	395	395	179	179	214	214	136	142
**383**	202	204	208	208	245	247	185	185	126	126	252	258	395	395	181	181	212	216	142	142
**384**	202	202	208	208	249	249	185	185	126	126	252	252	395	395	179	179	216	216	142	142
**385**	202	202	208	208	245	249	179	185	126	126	252	255	393	395	179	179	212	216	142	142
**386**	202	202	208	208	243	249	185	185	126	126	252	252	395	425	179	179	214	214	142	142
**387**	202	202	208	208	249	249	185	185	126	126	252	252	393	395	179	179	216	216	142	142
**388**	202	202	208	208	249	249	185	185	126	126	246	252	395	395	179	179	212	212	142	142
**389**	202	202	208	208	247	249	185	185	126	126	252	267	375	375	179	179	214	216	142	142
**390**	202	202	208	208	245	249	185	185	123	126	252	252	395	395	179	179	212	216	142	144
**391**	202	202	208	208	249	249	185	185	126	126	282	282	393	395	179	179	212	216	142	142
**392**	202	202	208	208	249	249	185	185	123	126	267	312	395	395	179	179	216	216	142	142
**393**	202	202	208	208	249	251	182	185	126	126	246	246	393	395	179	179	212	216	142	142
**394**	202	202	208	208	249	249	185	185	126	126	267	267	373	395	179	179	216	216	142	142
**395**	202	202	208	208	249	249	182	182	126	126	252	267	395	395	179	179	216	216	142	142
**396**	202	202	208	208	249	249	185	185	126	126	246	246	393	425	179	179	212	216	142	142
**397**	202	202	208	208	247	249	185	185	126	129	246	246	395	425	179	179	216	216	136	144
**398**	202	202	208	208	247	249	179	185	126	126	252	267	395	395	179	179	216	216	142	142
**399**	202	202	208	208	247	249	185	185	123	126	246	252	375	375	179	179	212	214	142	142
**400**	202	206	208	208	247	249	185	185	123	126	252	252	375	375	179	179	216	216	142	144
**401**	202	202	208	208	249	249	185	185	123	126	246	252	375	425	179	179	214	214	142	142
**402**	202	202	208	208	247	249	185	185	126	126	252	252	395	395	179	179	212	216	142	142
**403**	202	202	208	208	247	249	179	179	126	126	252	267	395	395	179	179	216	216	142	144
**404**	202	202	208	208	243	249	179	179	126	126	252	297	375	375	179	179	216	216	142	144
**405**	202	202	208	208	247	249	185	185	126	126	252	252	375	375	179	179	212	212	142	142
**406**	202	202	208	208	249	249	179	179	126	126	267	267	375	395	179	179	214	216	136	146
**407**	202	202	208	208	247	249	185	185	126	126	267	267	395	395	179	179	212	214	142	142
**408**	202	204	208	208	249	249	179	185	126	129	297	297	375	425	179	179	214	216	142	144
**409**	202	202	208	208	247	249	185	185	126	126	252	267	375	375	179	179	216	216	136	142
**410**	202	202	208	208	249	249	185	185	126	126	267	267	375	395	179	179	214	216	142	144
**411**	202	202	208	208	249	249	185	185	123	126			395	395	179	179	212	214	142	144
**412**	202	202	208	208	247	249	185	185	126	126	252	267	375	395	179	179	212	216	142	144
**413**	202	202	208	208	247	249	185	185	126	126	252	252	375	425	179	179	214	216	142	142
**414**	202	204	208	208	249	249	185	185	123	126	252	252	425	425	179	179	214	216	142	142
**415**	202	202	208	208	247	249	185	185	126	126	252	252	395	425	179	179	212	214	136	142
